# Supplementary material for: Apparent nonlinear damping triggered by quantum fluctuations
Source: Nat Commun. 2023 Nov 21;14:7566. doi: 10.1038/s41467-023-43128-y (PMC10663546; doi:10.1038/s41467-023-43128-y)
Supplement: Supplementary file 1 — Supplementary Information [file 41467_2023_43128_MOESM1_ESM.pdf]

Supplementary information for
“Apparent nonlinear damping triggered by quantum fluctuations”

Mario F. Gely,[1,3,*] Adrián Sanz Mora,[1] Shun Yanai,[1,4,5] Rik van der Spek,[1] Daniel Bothner,[1,2] and Gary A. Steele[1]

[1] *Kavli Institute of NanoScience, Delft University of Technology, PO Box 5046, 2600 GA, Delft, The Netherlands.*

[2] *Physikalisches Institut, Center for Quantum Science (CQ) and LISA$^+$, University of Tübingen, Auf der Morgenstelle 14, 72076 Tübingen, Germany*

[3] *Current address: Clarendon Laboratory, Department of Physics, University of Oxford, Parks Road, Oxford OX1 3PU, U.K.*

[4] *Current address: Institute for Quantum Computing, University of Waterloo, 200 University Avenue West, Waterloo, Ontario N2L 3G1, Canada*

[5] *Current address: Department of Physics and Astronomy, University of Waterloo, 200 University Avenue West, Waterloo, Ontario N2L 3G1, Canada*

(Dated: October 31, 2023)

SUPPLEMENTARY NOTE 1 – THEORETICAL DESCRIPTION

A Hamiltonian describing the series assembly of a inductor L, capacitor C and junction with Josephson inductance L_J can be entirely determined with only the knowledge of the admittance $Y(\omega) = 1/Z(\omega)$ across the Josephson junction, if we replace the latter by a linear inductor L_J. This approach assumes weak anharmonicity and damping of the circuit, and is often referred to as black-box quantization [1, 2]. The Hamiltonian writes

$$\hat{H}_\mathrm{bare}/\hbar = (\omega_\mathrm{r} - K)\, a^\dagger a - \frac{K}{2} a^\dagger a^\dagger a a \ , \tag{1}$$

where ω_r satisfies $Y(\omega_\mathrm{r}) = 0$

$$\omega_\mathrm{r} = \frac{1}{\sqrt{(L + L_\mathrm{J})C}} \tag{2}$$

and the Kerr constant is given by

$$\hbar K = \frac{2e^2}{L_\mathrm{J}\omega_\mathrm{r}^2(\mathrm{Im}Y'(\omega_\mathrm{r}))^2} = \frac{e^2}{2C}\left(\frac{L_\mathrm{J}}{L + L_\mathrm{J}}\right)^3 \ . \tag{3}$$

The weak anharmonicity assumption which leads to this Hamiltonian writes $K \ll \omega_\mathrm{r}$.

This circuit loses energy through resistive losses at a rate κ_int, and can exchange energy with a transmission line at a rate κ_ext. The total rate at which the circuit loses energy is then $\kappa = \kappa_\mathrm{int} + \kappa_\mathrm{ext}$. On one end of the transmission line, we feed a coherent signal with power P_in oscillating at ω_d. Following quantum input-output theory [3], the dynamics of $\hat{a}(t)$, in a frame rotating at ω_d, is given by

$$\frac{d}{dt}\hat{a}(t) = -i\left(\Delta - K\hat{a}(t)^\dagger\hat{a}(t)\right)\hat{a}(t) - \frac{\kappa}{2}\hat{a}(t) + \epsilon - \sqrt{\kappa}\hat{s} \ , \tag{4}$$

where $\Delta = \omega_\mathrm{r} - K - \omega_\mathrm{d}$, and the strength of the drive is characterized by $\epsilon = \sqrt{\kappa_\mathrm{ext} P_\mathrm{in}/(2\hbar\omega_\mathrm{r})}$. Note the factor 2 in the denominator which corresponds to the fact that there are two directions of propagation in the feedline and that

* Corresponding author, mario.gely@physics.ox.ac.uk

only one is occupied by the driving signal. The term $\hat{s}$ corresponds to both thermal noise and quantum vacuum noise. We assume it to be well described by quantum white noise, a stationary random process which is characterized by its 0 mean $\langle \hat{s} \rangle = 0$ and the correlation functions

$$\begin{aligned} \langle \hat{s}(t)\hat{s}^\dagger(t') \rangle &= \langle \hat{s}^\dagger(t')\hat{s}(t) \rangle + \delta(t-t') = [n_{\rm th}+1]\delta(t-t') \\ \langle \hat{s}(t)\hat{s}(t') \rangle &= \langle \hat{s}^\dagger(t')\hat{s}^\dagger(t) \rangle = 0 \ . \end{aligned} \tag{5}$$

Here $n_{\rm th}$ corresponds to the average number of excitations induced by the thermal environment at a temperature T

$$n_{\rm th} = \frac{1}{e^{\frac{\hbar\omega_{\rm r}}{k_{\rm B}T}} - 1} \ , \tag{6}$$

with $k_{\rm B}$ corresponding to Boltzmann's constant, to which is added "+1" corresponding to the quantum vacuum fluctuations. As described in Supplementary Note 7, thermal noise is heavily attenuated such that the resonator has a thermal occupation $n_{\rm th} < 0.05$, a much smaller source of noise than quantum fluctuations. We may thus safely make the approximation $n_{\rm th} \simeq 0$ when describing the experiment. The S_{21} parameter is obtained from $\langle \hat{a}(t) \rangle$ as

$$S_{21} = 1 - \frac{\kappa_{\rm ext}}{2\epsilon}\langle \hat{a} \rangle \ , \tag{7}$$

the factor 2 again reflecting that only half of the signal emitted by the circuit will travel towards the receiver of the VNA. As an alternative to the Langevin equation, one may also formulate the problem in terms of a Lindblad master equation [4]

$$\begin{aligned} \frac{\partial \hat{\rho}}{\partial t} = &- i\left[\Delta \hat{a}^\dagger \hat{a} - \frac{K}{2}\hat{a}^\dagger\hat{a}^\dagger\hat{a}\hat{a} + i\epsilon(\hat{a}^\dagger - \hat{a}), \hat{\rho}\right] \\ &+ \kappa(n_{\rm th}+1)D(\hat{a})\hat{\rho} + \kappa n_{\rm th} D(\hat{a}^\dagger)\hat{\rho} \ , \end{aligned} \tag{8}$$

governing the density matrix of the system $\hat{\rho}$, where

$$D(\hat{L})\hat{\rho} = \hat{L}\hat{\rho}\hat{L}^\dagger - \{\hat{L}\hat{L}^\dagger, \hat{\rho}\}/2 \ . \tag{9}$$

SUPPLEMENTARY NOTE 2 – DATA PROCESSING AND FITTING

Following the approach described in the Methods section, excellent agreement between the numerical computation based on the Lindblad equation and the experimental data is found for the complex transmission at all drive powers, as well as for the drive power dependence of both the minimum value of $|S_{21}|$ and the driving frequency at which this minimum is reached, see Supplementary Fig. 1.

The fitted parameters are confirmed by the measurement of a reference oscillator [5], built using the same geometry as the device in Fig. 1 (and in the same fabrication run) but where the SQUID is replaced by a short circuit. We simulate the resonance frequency of the reference oscillator using the finite-element software Sonnet, and compare it to the experimentally measured value. The discrepancy between these allows us to determine the kinetic inductance of the 60 nm sheet of MoRe. Using a sheet inductance of 1.575 pH/sq in Sonnet, the simulated resonance frequency matches the measured value. We then add a lumped element inductor $L_{\rm J}$ at the location of the SQUID in the simulation, and vary it to determine the value of the lumped element inductor L and capacitor C, by fitting simulated resonance frequencies to $1/\sqrt{(L+L_{\rm J})C}$. The Josephson inductance $L_{\rm J}$ is found when this simulated resonance matches the low-power resonance frequency measured in Fig. 2. The circuit parameters obtained from this analysis are $L = 2.93$ nH, $C = 288$ fF and $L_{\rm J} = 0.35$ nH. The resonance frequency and Kerr constant calculated from these parameters matches those determined by fitting the data with small deviations of 0.1% and 2% respectively.

Finally, we would like to acknowledge that further measurements could have been performed to validate both the system parameters and the physical mechanism of apparent non-linear damping. The Kerr constant could be verified by strongly driving the system off-resonance and measuring the resulting Stark shift with a low-power probe. The effective non-linear damping parameter γ could be verified through the change in S_{11} expected from a varied thermal occupation $n_{\rm th}$. Here thermal occupation could be varied by injecting a known white noise or by increasing the base temperature of the dilution refrigerator. Lastly, the SQUID tunability could be used to demonstrate an understanding of the apparent non-linear damping with a different Kerr constant and resonator frequency. Unfortunately, such measurements can no longer be carried out in a practical timescale: the device was fabricated for a different purpose and measured long before the analysis presented this work was performed and is no longer in working condition.

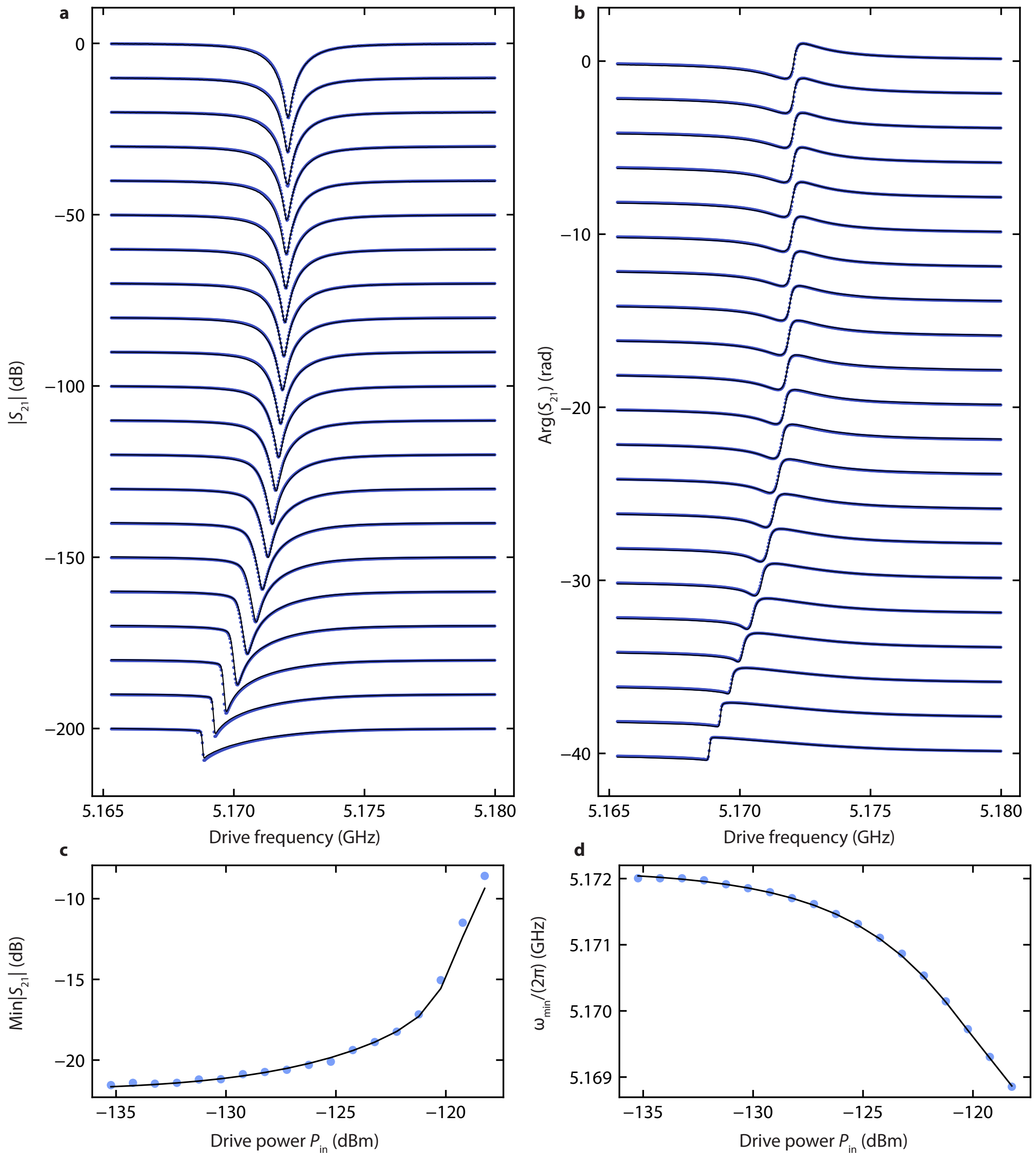

Supplementary Fig. 1. **Transmission coefficient of the superconducting resonant circuit.** Measurements of the transmission coefficient S_{21}, processed as described in the Methods section, are used in a curve fitting routine that seeks, for each driving power in the dataset and $n_{\text{th}} = 0$, a numerical solution of the steady-state equation (Supp. Eq. (8)) of the damped driven quantum Kerr oscillator with the set of oscillator parameters that fits best to the measured data in accordance to a minimization of the error in: **a,** the magnitude $|S_{21}|$ and **b,** phase $\text{Arg}(S_{21})$ of S_{21} as a function of driving frequency, as well as in **c,** the minimum $\text{Min}|S_{21}|$ of $|S_{21}|$ and **d,** the driving frequency $\omega_{\min}$ at which this minimum occurs as a function of drive power. Data-points are rendered with blue dots and curve fits with solid lines. Each trace in **a** and **b** corresponds to a different drive power. At the top trace the drive power is $P_{\text{in}} = -135.23$ dBm, then this increases 1 dBm per trace, reaching $P_{\text{in}} = -118.23$ dBm at the bottom trace. We offset every trace in **a** and **b** by -10 dB and -2 rad respectively to ease examination.

SUPPLEMENTARY NOTE 3 – CLASSICAL, NOISE-LESS SOLUTION AND BIFURCATION POINT

Taking the expectation value of Supp. Eq. (4), in the steady-state ($d\langle \hat{a} \rangle/dt = 0$), yields

$$i\Delta\langle \hat{a} \rangle - iK\langle \hat{a}^\dagger \hat{a}\hat{a} \rangle + \frac{\kappa}{2}\langle \hat{a} \rangle = \epsilon \ . \tag{10}$$

In the absence of (thermal and quantum) noise $\hat{s}=0$, using the notation $\langle\hat{a}\rangle = a$, one can simply write $\langle\hat{a}^\dagger\hat{a}\hat{a}\rangle = |a|^2 a$. This yields the classical steady-state equation

$$\left(i\Delta - iK|a|^2 + \frac{\kappa}{2}\right) a = \epsilon \ . \tag{11}$$

Note that $\Delta = \omega_\mathrm{r} - \omega_\mathrm{d}$ in the classical case, as the extra $-K$ in the definition of Δ in the quantum Langevin equation (Supp. Eq. (4)) is a consequence of the quantum fluctuations similar to the Lamb shift [6]. One can see this by rewriting the Kerr term $\hat{a}^\dagger\hat{a}^\dagger\hat{a}\hat{a}$ using the commutation relations, which would yield a different expression for Δ in the quantum equation, but would not yield a different Kerr term in the classical steady-state equation.

To simulate nonlinear damping, we consider that the internal damping rate can depend on power $\kappa_\mathrm{int} \to \kappa_\mathrm{int} + \gamma|a|^2$ yielding

$$\left(i\Delta - iK|a|^2 + \frac{\kappa + \gamma|a|^2}{2}\right) a = \epsilon \ . \tag{12}$$

We solve this equation by computing the magnitude $|a|$ and phase φ of the amplitude $a = |a|e^{i\varphi}$ separately. An equation for $|a|$ is obtained by multiplying Supp. Eq. (11) by its conjugate, yielding

$$\left(\frac{\gamma^2}{4} + K^2\right)|a|^6 + \left(\frac{\gamma\kappa}{2} - 2\Delta K\right)|a|^4 + \left(\frac{\kappa^2}{4} + \Delta^2\right)|a|^2 = \epsilon^2 \ . \tag{13}$$

We solve this equation by computing the eigenvalues of the polynomial's companion matrix [7] using Python [8]. The phase is then given by

$$\varphi = \arctan\left(\frac{2(\Delta - K|a|^2)}{\kappa + \gamma|a|^2}\right) . \tag{14}$$

Beyond a critical drive power there are drive frequencies for which Supp. Eq. (13) possesses not only one but three real solutions. In that case, the steady-state response of the oscillator will exhibit bistability as the drive frequency or detuning is varied. The point (detuning) at which the onset of bistability takes place is known as a critical point (detuning). The same applies to Supp. Eq. (13) with $\gamma = 0$, in which case the ensuing critical power $P_\mathrm{c} = 2\sqrt{3}\hbar\omega_\mathrm{r}\kappa^3/(9|K|\kappa_\mathrm{ext}) \simeq -122.2$ dBm is obtained through a simple stability analysis, see e.g. Appendix C of Ref. [9]. In what follows, we shall then use P_c as an estimate of the actual critical power that follows with $\gamma \neq 0$. An examination of Supplementary Figs. 1(a,b), suggests that this is a reasonable approximation.

Most of our measurements employ drive powers lower than P_c. Furthermore, we observe that the phenomenon we are concerned with, namely, the reduction in the oscillator's amplitude as the drive power increases, manifests clearly in all those measurements. Either a fully classical description of a steady-state in a nonlinear quantum system (the superconducting resonant circuit in our case) or that based on a linearization of quantum fluctuations around such a steady-state, are accurate far from a critical point. However, both descriptions are known to fail in the vicinity of a critical point, see Ref. [10] for more details on this regard. As a consequence, we focus all the theoretical analysis we present in this work on a frequency response of the resonant circuit describable by a steady-state oscillator well below the bistability threshold. To ensure this condition, we only consider drive powers $P_\mathrm{in} \leq -124$ dBm, further motivated in Supplementary Note 4.

Bearing in mind the above, we now search for the largest attainable amplitude for a given driving strength in Supp. Eq. (13) by assuming a small deviation from the largest attainable amplitude without nonlinearities

$$\begin{aligned} |a| &= \alpha(1+\delta) \ , \\ |a|^n &\simeq \alpha^n(1+n\delta) \end{aligned} \tag{15}$$

with $\delta \ll 1$. The amplitude α is the solution to

$$-\frac{\kappa}{2}a + \epsilon = 0 \ , \tag{16}$$

the resonantly driven system, without nonlinearities. Assuming a small deviation from α means we are considering the nonlinearities K, γ as well as the detuning Δ to be perturbations, such that

$$K, \gamma, \Delta \ll \kappa \ . \tag{17}$$

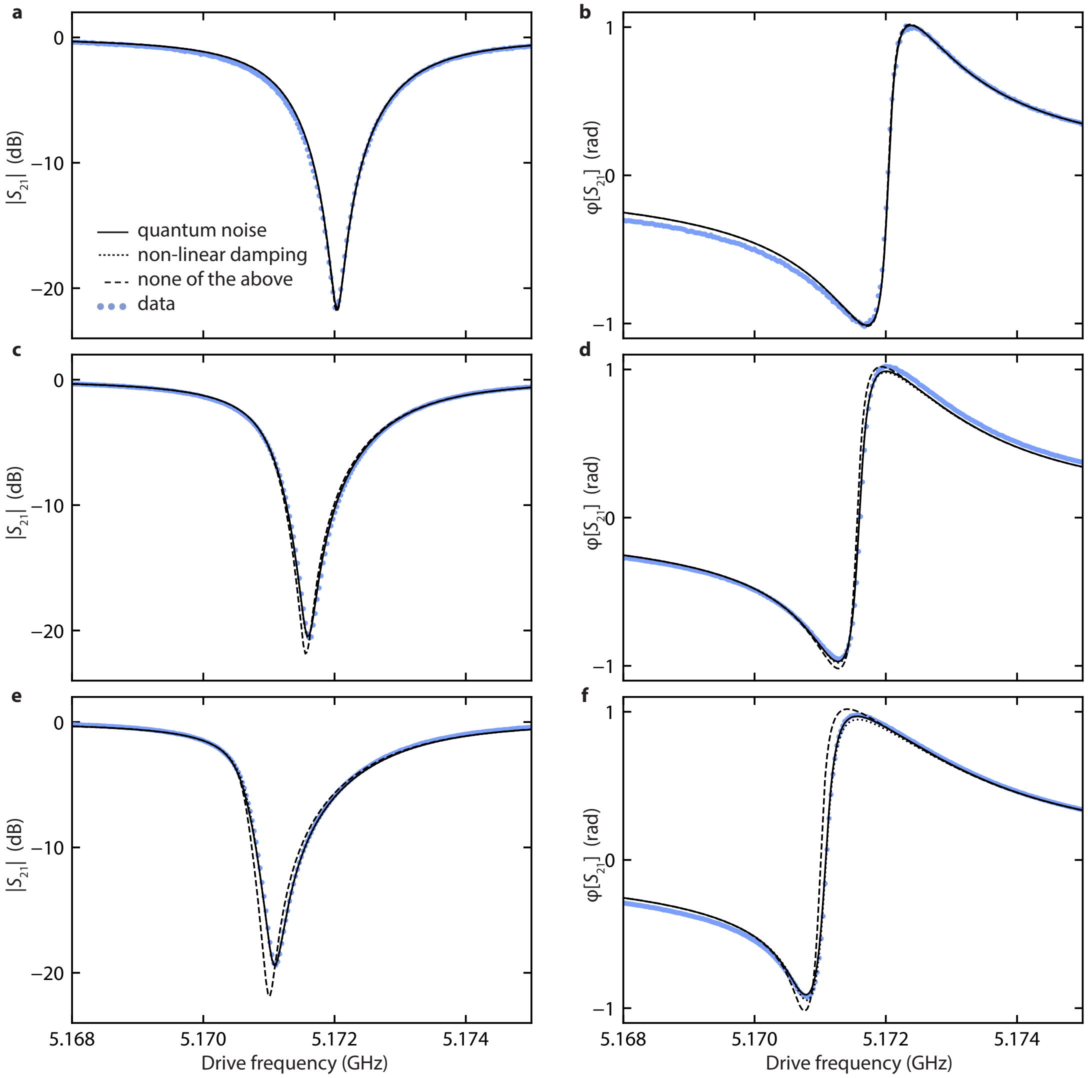

Supplementary Fig. 2. **Quantum vs. classical descriptions: transmission coefficient.** Data-points (blue dots) of the transmission coefficient S_{21}, processed following Supplementary Note 2, are compared to the best curve fits resulting from both a numerical solution of the Lindblad equation (Supp. Eq. (8)) (solid line), and a numerical solution of the classical steady-state equation (Supp. Eq. (12)) with nonlinear damping (dotted line), following, respectively, the minimization routines described in Supplementary Note 2 and Supplementary Note 3. We also display the solution to the steady-state equation (Supp. Eq. (11)) of the linearly damped driven classical Kerr oscillator (dashed line). **a, c, e** The magnitude $|S_{21}|$ and **b, d, f** phase $\varphi[S_{21}] = \mathrm{Arg}(S_{21})$ of the transmission coefficient are plotted as a function of the drive frequency. Panels (a,b), (c,d) and (e,f) correspond to powers -135 dBm, -127 dBm and -124 dBm respectively; all of them associated with a steady-state oscillator below the bistability threshold.

Since $\Delta \simeq K\alpha^2$, we note that these approximations are only valid at the lower powers of our experimental data. Injecting the perturbed expression for $|a|$ into Supp. Eq. (13), we obtain an equation for δ

$$\left(\gamma^2/4 + K^2\right)\alpha^6(1+6\delta) + \left(\gamma\kappa/2 - 2\Delta K\right)\alpha^4(1+4\delta) + \left(\kappa^2/4 + \Delta^2\right)\alpha^2(1+2\delta) = \epsilon^2 \; . \tag{18}$$

Expanding the solution

$$\delta = \frac{\epsilon^2 - \left(\gamma^2/4 + K^2\right)\alpha^6 - \left(\gamma\kappa/2 - 2\Delta K\right)\alpha^4 - \left(\kappa^2/4 + \Delta^2\right)\alpha^2}{6\left(\gamma^2/4 + K^2\right)\alpha^6 + 4\left(\gamma\kappa/2 - 2\Delta K\right)\alpha^4 + 2\left(\kappa^2/4 + \Delta^2\right)\alpha^2} \; , \tag{19}$$

to second order in K, γ, Δ through the approximation of Supp. Eq. (17) yields

$$\delta \simeq -\frac{\alpha^2\gamma}{\kappa} - \frac{4K^2\alpha^4 - 7\alpha^4\gamma^2 - 8K\alpha^2\Delta + 4\Delta^2}{2\kappa^2} \tag{20}$$

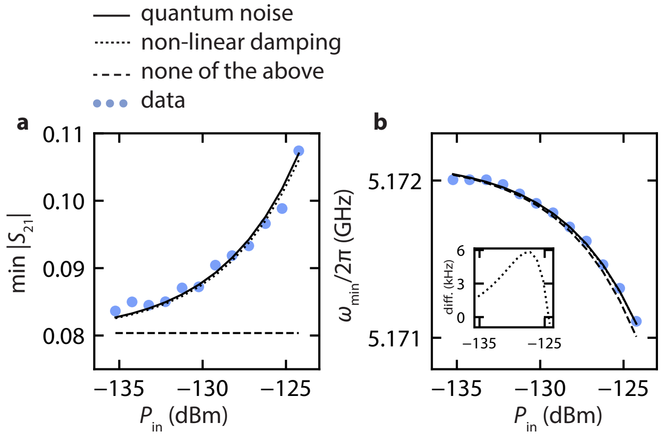

Supplementary Fig. 3. **Quantum vs. classical descriptions: minimum of $|S_{21}|$.** **a**, The minimum Min$|S_{21}|$ of transmission $|S_{21}|$ and **b**, the frequency $\omega_{\min}$ which minimizes it, are plotted as a function of the drive power. We obtain excellent agreement between experimental data (dots), the fit based on a numerical solution of the Lindblad master equation (solid line), as described in Supplementary Note 2, and the fit based on a numerical solution of the classical nonlinear damping model (dotted line), as described in Supplementary Note 3. On the contrary, in **a** the minimum of transmission $|1-\kappa_{\rm ext}/\kappa|$ that follows from the solution to the classical steady-state equation (Supp. Eq. (11)) with linear damping (dashed line) contrasts sharply with the experimental data. In **b**, due to the overlap between the solid and dotted lines, the inset shows their difference as a function of drive power. The drive power is bound to $P_{\rm in} \leq -124$ dBm, in which case the steady-state of the oscillator lies below the bistability threshold.

which is minimized for

$$\Delta = K\alpha^2 \tag{21}$$

with a maximum (to leading order in γ)

$$\delta = -\frac{\alpha^2\gamma}{\kappa} \ . \tag{22}$$

In order to demonstrate that a classical nonlinear damping model also provides an adequate description of the data, we fit the data to a solution to Supp. Eq. (12). The data processing, and the construction of the cost function, is identical to that described in Supplementary Note 2. The free-parameters, however, are different: we fix the internal and external damping, the oscillator frequency to $\omega_r - K$ and the attenuation to the values fitted with the quantum model. The remaining free parameters of the fit are the nonlinear damping rate γ, and the Kerr nonlinearity K. After convergence of the same modified Powell algorithm [8, 11], we obtain $\gamma = 2\pi \times 5.02$ kHz and $K = 2\pi \times 78.3$ kHz (1.7 kHz lower than the value obtained by fitting the quantum model). In Supplementary Figs. 2,3, we show that this model provides a reasonable fit to the data we address in our theoretical description (below the bistability threshold of the steady-state of our model oscillator).

SUPPLEMENTARY NOTE 4 – QUANTUM AND NOISY SOLUTION IN THE GAUSSIAN REGIME

Here we derive an approximate solution to the Langevin equation of Supp. Eq. (4) in the presence of quantum and/or thermal noise. The aim is to solve the equation for the ensemble average $\langle\hat{a}\rangle$

$$\frac{d}{dt}\langle\hat{a}\rangle = -[i\Delta + \frac{\kappa}{2}]\langle\hat{a}\rangle + iK\langle\hat{a}^\dagger\hat{a}^2\rangle + \epsilon \ , \tag{23}$$

in the steady-state and in a small neighborhood of the resonance. In order to lighten our notation, we drop the time dependency in the equations and, unless it is explicitly stated otherwise, identify the ensuing dynamical variables with those that define the steady-state of the system. Contrary to the noise-less case studied in the previous section, here $\langle\hat{a}^\dagger\hat{a}\hat{a}\rangle \neq |a|^2 a$. This nonlinear term in Supp. Eq. (23) above leads to a Bogoliubov-Born-Green-Kirkwood-Yvon (BBGKY) like hierarchy of coupled differential equations, in which, e.g, the dynamics of the moment $\langle\hat{a}^n\rangle$ of order

n requires knowledge of the higher order moment $\langle \hat{a}^\dagger a^{n+1} \rangle$, with $n \in \mathbb{N}^+$ [12]. To perform a tractable analytical study of such infinite hierarchy of equations we shall truncate it appropriately. We do so using a Gaussian state approximation which allows for closing the hierarchy, thus guaranteeing a physically meaningful steady-state solution [10, 12, 13]. The resulting closed system of coupled differential equations involves only first order statistical moments (*i.e.* ensemble averages) and second order statistical moments (covariances) of the system's canonical operators. This approach, different to assuming a coherent state ansatz for the oscillator's quantum state, does not prescribe an operator's ensemble average to be entirely ruled by the classical dynamics of the system. Instead, in this case an operator's ensemble average is treated as an unknown variable to be determined self-consistently with its covariances, thus including some information of the quantum dynamics of the system into that of the operator's ensemble averages. Next, we present the conditions under which we may apply such an approach, followed by the description of a method for obtaining the ensemble average $\langle \hat{a} \rangle$ resulting from our Gaussian state ansatz.

A. Assumptions and small quantities

We assume that the thermal fluctuations, as well as the nonlinearity, have a small effect on the system. This approximation comes in two forms. First, we will assume the thermal fluctuations entering through the noise term $\hat{s}$ to be small through the condition

$$n_{\text{th}} \ll |\langle \hat{a} \rangle|^2 \ . \tag{24}$$

Secondly, we will assume the anharmonicity to be small, through the condition

$$K \ll \kappa \ . \tag{25}$$

In this regime, it is practical to express the equations using the deviation $\hat{d}$ from the steady-state expectation values of the operators involved in the dynamics. For $\hat{a}$, this means we will write

$$\hat{a} = \langle \hat{a} \rangle + \hat{d} \ . \tag{26}$$

We will take $\hat{d}$ to be a small quantity through the assumption

$$\Big|\text{Re}\big[\langle \hat{d}^{\dagger n} \hat{d}^m \rangle\big]\Big| \ll \Big|\text{Re}\big[\langle \hat{a}^{\dagger n} \hat{a}^m \rangle - \langle \hat{d}^{\dagger n} \hat{d}^m \rangle\big]\Big| \text{ and } \Big|\text{Im}\big[\langle \hat{d}^{\dagger n} \hat{d}^m \rangle\big]\Big| \ll \Big|\text{Im}\big[\langle \hat{a}^{\dagger n} \hat{a}^m \rangle - \langle \hat{d}^{\dagger n} \hat{d}^m \rangle\big]\Big| \text{ if } n+m > 2 \,, \tag{27}$$

such that we will neglect $\langle \hat{d}^{\dagger n} \hat{d}^m \rangle$ with respect to $\langle \hat{a}^{\dagger n} \hat{a}^m \rangle - \langle \hat{d}^{\dagger n} \hat{d}^m \rangle$ and thus use $\langle \hat{d}^{\dagger n} \hat{d}^m \rangle \simeq 0$ whenever $n+m > 2$. This second order ladder approximation amounts to considering that the steady-state is well described by a Gaussian state. We note that for a Gaussian state $\langle (\hat{d}^\dagger \hat{d})^m \rangle$ scales with n_{th}^m. Therefore, considering that the thermal fluctuations are small $n_{\text{th}} \ll |\langle \hat{a} \rangle|^2$ is necessary for Supp. Eq. (27) to hold.

The assumption (27) enables a linearization of the steady-state equations of the ensemble averages and covariances of the oscillator's canonical operators that fully characterize the resulting Gaussian steady-state. As shown for a similar paradigm in Ref. [13], given a limit of weak anharmonicity, c.f. Supp. Eq. (25), and far from a multistability threshold, this linearization leads to an analytical steady-state solution in good agreement with numerical calculations. This approach is then applicable for our system below the bistability threshold. Although lacking the precision of other methods [14, 15], it provides a faithful description of our experimental observations, with the additional benefit that quantities of interest (such as averages and covariances of canonical operators) can be expressed more simply.

B. Preliminary results

We first derive expressions for useful expectation values. Injecting Supp. Eq. (26) in $\langle \hat{a}^\dagger \hat{a} \rangle$ for example yields

$$\langle \hat{a}^\dagger \hat{a} \rangle = \Big\langle (\langle \hat{a} \rangle^* + \hat{d}^\dagger)(\langle \hat{a} \rangle + \hat{d}) \Big\rangle \tag{28}$$

$$= \langle \hat{a} \rangle^* \langle \hat{a} \rangle + \langle \hat{a} \rangle^* \langle \hat{d} \rangle + \langle \hat{a} \rangle \langle \hat{d}^\dagger \rangle + \langle \hat{d}^\dagger \hat{d} \rangle \tag{29}$$

$$= \langle \hat{a} \rangle^* \langle \hat{a} \rangle + \langle \hat{d}^\dagger \hat{d} \rangle \ , \tag{30}$$

where we have used the definition of $\hat{d}$ to obtain $\langle\hat{d}\rangle = \langle\hat{d}^\dagger\rangle = 0$. We proceed similarly to obtain

$$\langle\hat{a}^2\rangle = \langle\hat{a}\rangle^2 + \langle\hat{d}^2\rangle \ , \tag{31}$$

and by invoking the approximation of Supp. Eq. (27), we also have

$$\langle\hat{a}^\dagger\hat{a}^2\rangle \simeq 2\langle\hat{d}^\dagger\hat{d}\rangle\langle\hat{a}\rangle + \langle\hat{a}^\dagger\rangle\langle\hat{d}^2\rangle + |\langle\hat{a}\rangle|^2\langle\hat{a}\rangle \ , \tag{32}$$

$$\langle\hat{a}^\dagger\hat{a}^3\rangle \simeq |\langle\hat{a}\rangle|^2\langle\hat{a}\rangle^2 + 3|\langle\hat{a}\rangle|^2\langle\hat{d}^2\rangle + 3\langle\hat{a}\rangle^2\langle\hat{d}^\dagger\hat{d}\rangle \ . \tag{33}$$

C. Reformulation of the problem

By injecting Supp. Eq. (32) in Supp. Eq. (23), we rewrite the equation for $\langle\hat{a}\rangle$ as

$$\frac{d}{dt}\langle\hat{a}\rangle \simeq -\left[i(\Delta - 2K\langle\hat{d}^\dagger\hat{d}\rangle - K|\langle\hat{a}\rangle|^2) + \kappa/2\right]\langle\hat{a}\rangle + iK\langle\hat{a}^\dagger\rangle\langle\hat{d}^2\rangle + \epsilon. \tag{34}$$

A steady-state solution for $\langle\hat{a}\rangle$ thus requires knowledge of $\langle\hat{d}^\dagger\hat{d}\rangle$ and $\langle\hat{d}^2\rangle$. These expectation values will be determined by deriving the equation of motion of $\hat{d}^\dagger\hat{d}$ and $\hat{d}^2$.

D. Equation of motion for $\langle\hat{d}^\dagger\hat{d}\rangle$

From Supp. Eq. (30), we have $\langle\hat{d}^\dagger\hat{d}\rangle = \langle\hat{a}^\dagger\hat{a}\rangle - \langle\hat{a}\rangle^*\langle\hat{a}\rangle$ such that

$$\frac{d}{dt}\langle\hat{d}^\dagger\hat{d}\rangle = \frac{d}{dt}\langle\hat{a}^\dagger\hat{a}\rangle - \langle\hat{a}\rangle^*\frac{d}{dt}\langle\hat{a}\rangle - \langle\hat{a}\rangle\left(\frac{d}{dt}\langle\hat{a}\rangle\right)^* \ . \tag{35}$$

We should thus search for the equation of motion for $\langle\hat{a}^\dagger\hat{a}\rangle$. Utilizing the Langevin equation of Supp. Eq. (4), we first obtain an equation for $\hat{a}^\dagger\hat{a}$

$$\frac{d}{dt}\left(\hat{a}^\dagger\hat{a}\right) = \left(\frac{d}{dt}\hat{a}^\dagger\right)\hat{a} + \hat{a}^\dagger\left(\frac{d}{dt}\hat{a}\right) \tag{36}$$

$$= -\kappa\hat{a}^\dagger\hat{a} + \epsilon(\hat{a} + \hat{a}^\dagger) - \sqrt{\kappa}(\hat{a}^\dagger\hat{s} + \hat{s}^\dagger\hat{a}) \ . \tag{37}$$

When taking the expectation value of this equation, we follow Ref. [16] to treat the noise terms. For $\hat{s}$ given by quantum noise, with $\langle\hat{s}\rangle = 0$ and Supp. Eqs. (5), then given $\hat{A}$ an arbitrary system operator, we have

$$\langle\hat{A}(t)\hat{s}(t)\rangle = n_{\rm th}\frac{\sqrt{\kappa}}{2}\ \langle[\hat{A}(t), \hat{a}(t)]\rangle \ , \tag{38}$$

$$\langle\hat{s}^\dagger(t)\hat{A}(t)\rangle = n_{\rm th}\frac{\sqrt{\kappa}}{2}\ \langle[\hat{a}^\dagger(t), \hat{A}(t)]\rangle \ , \tag{39}$$

such that $\langle\hat{a}^\dagger\hat{s} + \hat{s}^\dagger\hat{a}\rangle = -\sqrt{\kappa}n_{\rm th}$. Using Supp. Eq. (30) again to rewrite $\langle\hat{a}^\dagger\hat{a}\rangle$ we have

$$\frac{d}{dt}\langle\hat{a}^\dagger\hat{a}\rangle = -\kappa(|\langle\hat{a}\rangle|^2 + \langle\hat{d}^\dagger\hat{d}\rangle) + \epsilon(\langle\hat{a}\rangle + \langle\hat{a}^\dagger\rangle) + \kappa n_{\rm th} \ . \tag{40}$$

Using the equation of motion for $\langle\hat{a}\rangle$ of Supp. Eq. (34), we finally obtain

$$\frac{d}{dt}\langle\hat{d}^\dagger\hat{d}\rangle = \frac{d}{dt}\langle\hat{a}^\dagger\hat{a}\rangle - \langle\hat{a}\rangle^*\frac{d}{dt}\langle\hat{a}\rangle - \langle\hat{a}\rangle\left(\frac{d}{dt}\langle\hat{a}\rangle\right)^* \tag{41}$$

$$\simeq -\kappa\langle\hat{d}^\dagger\hat{d}\rangle + \kappa n_{\rm th} + iK\left(\langle\hat{d}^{\dagger 2}\rangle\langle\hat{a}\rangle^2 - \langle\hat{a}^\dagger\rangle^2\langle\hat{d}^2\rangle\right) \ . \tag{42}$$

E. Equation of motion for $\langle \hat{d}^2 \rangle$

From Supp. Eq. (31) we have $\langle \hat{d}^2 \rangle = \langle \hat{a}^2 \rangle - \langle \hat{a} \rangle^2$, such that the equation of motion for $\langle \hat{d}^2 \rangle$ writes

$$\frac{d}{dt}\langle \hat{d}^2 \rangle = \frac{d}{dt}\langle \hat{a}^2 \rangle - 2\langle \hat{a} \rangle \left(\frac{d}{dt}\langle \hat{a} \rangle \right) \ . \tag{43}$$

We should thus search for the equation of motion for $\langle \hat{a}^2 \rangle$. We start by writing the equation of motion for $\hat{a}^2$ as

$$\begin{aligned} \frac{d}{dt}\hat{a}^2 &= \hat{a}\left(\frac{d}{dt}\hat{a} \right) + \left(\frac{d}{dt}\hat{a} \right)\hat{a} \\ &= -i\left(2\Delta - K\{\hat{a}^\dagger, \hat{a}\}\right)\hat{a}^2 - \kappa \hat{a}^2 + 2\epsilon \hat{a} - \sqrt{\kappa}\left(\hat{s}\hat{a} + \hat{a}\hat{s}\right) \ . \end{aligned} \tag{44}$$

If quantum noise is taken into account, then we have $\{\hat{a}^\dagger, \hat{a}\} = 1 + 2\hat{a}^\dagger \hat{a}$ rather than $\{a^*, a\} = 2a^* a$ without. When taking the expectation values, we treat the noise terms in $\hat{s}$ following Supp. Eq. (38), and an additional result from Ref. [16]

$$\langle \hat{s}(t)\hat{A}(t) \rangle = (n_{\text{th}} + 1)\frac{\sqrt{\kappa}}{2} \langle [\hat{A}(t), \hat{a}(t)] \rangle \ , \tag{45}$$

resulting in $\langle \hat{s}\hat{a} + \hat{a}\hat{s} \rangle = 0$. By rewriting $\langle \hat{a}^2 \rangle$ using Supp. Eq. (31) and $\langle \hat{a}^\dagger \hat{a}^3 \rangle$ using Supp. Eq. (33), we get

$$\frac{d}{dt}\langle \hat{a}^2 \rangle \simeq 2iK\left(|\langle \hat{a} \rangle|^2 \langle \hat{a} \rangle^2 + 3|\langle \hat{a} \rangle|^2 \langle \hat{d}^2 \rangle + 3\langle \hat{a} \rangle^2 \langle \hat{d}^\dagger \hat{d} \rangle \right) - (2i\Delta - iK + \kappa)\left(\langle \hat{a} \rangle^2 + \langle \hat{d}^2 \rangle \right) + 2\epsilon \langle \hat{a} \rangle \ . \tag{46}$$

Using this equation as well as the equation of motion for $\langle \hat{a} \rangle$ of Supp. Eq. (34), we finally obtain

$$\frac{d}{dt}\langle \hat{d}^2 \rangle = \frac{d}{dt}\langle \hat{a}^2 \rangle - 2\langle \hat{a} \rangle \left(\frac{d}{dt}\langle \hat{a} \rangle \right) \tag{47}$$

$$\simeq -2\left(i\left(\Delta - 2K(|\langle \hat{a} \rangle|^2 + \frac{1}{4}) \right) + \kappa/2 \right)\langle \hat{d}^2 \rangle + i2K\langle \hat{a} \rangle^2 \left(\langle \hat{d}^\dagger \hat{d} \rangle + \frac{1}{2} \right) \ . \tag{48}$$

F. Steady-state solution

We obtain a steady-state solution for the equations of motion (34,42,48) by equating all time derivatives to zero. We first solve for the covariances to get

$$\langle \hat{d}^\dagger \hat{d} \rangle \simeq \frac{n_{\text{th}} + \frac{1}{2}\frac{4K^2|\langle \hat{a} \rangle|^4}{4\Omega^2 + \kappa^2}}{1 - \frac{4K^2|\langle \hat{a} \rangle|^4}{4\Omega^2 + \kappa^2}} = \frac{n_{\text{th}} + \frac{1}{2}}{1 - \frac{4K^2|\langle \hat{a} \rangle|^4/\kappa^2}{4\Omega^2/\kappa^2 + 1}} - \frac{1}{2}, \tag{49}$$

$$\langle \hat{d}^2 \rangle \simeq i\frac{K\langle \hat{a} \rangle^2}{i\Omega + \kappa/2}\left(\langle \hat{d}^\dagger \hat{d} \rangle + \frac{1}{2} \right) = \frac{4K}{\kappa}\langle \hat{a} \rangle^2 \frac{n_{\text{th}} + \frac{1}{2}}{4\Omega^2/\kappa^2 - 4K^2|\langle \hat{a} \rangle|^4/\kappa^2 + 1}(\Omega/\kappa + i/2) \tag{50}$$

where $\Omega = \Delta - K(2|\langle \hat{a} \rangle|^2 + 1/2)$. By injecting our expressions for $\langle \hat{d}^\dagger \hat{d} \rangle, \langle \hat{d}^2 \rangle$ (c. f. Supp. Eqs. (49) and (50), respectively) in Supp. Eq. (34) and after some algebra, we find that, in the steady-state, the amplitude is ruled by the following equation

$$\begin{aligned} &i\left[\left(\Delta - \frac{K}{2} \right)\left(1 - \frac{\gamma|\langle \hat{a} \rangle|^2/\kappa}{4\Omega^2/\kappa^2 - 4K^2|\langle \hat{a} \rangle|^4/\kappa^2 + 1} \right) + \frac{3K}{2} - K|\langle \hat{a} \rangle|^2 - 2K\left(n_{\text{th}} + \frac{1}{2} \right) \right]\langle \hat{a} \rangle \\ &+ \frac{\kappa}{2}\left[1 + \frac{\gamma|\langle \hat{a} \rangle|^2/\kappa}{4\Omega^2/\kappa^2 - 4K^2|\langle \hat{a} \rangle|^4/\kappa^2 + 1} \right]\langle \hat{a} \rangle \simeq \epsilon \ . \end{aligned} \tag{51}$$

Supp. Eq. (51) shows that the interplay of the oscillator's nonlinearity and the noise gives rise, among other effects, to a nonlinear broadening of the oscillator's steady-state response, the strength of which is given by

$$\gamma = \frac{4K^2}{\kappa}\left(n_{\text{th}} + \frac{1}{2} \right) \ . \tag{52}$$

These effects resulting from the interplay of the noise and the oscillator's nonlinearity manifest most prominently as we approach to a resonance scenario, for if, on the contrary $|\Delta/\kappa| \rightarrow \infty$, the distribution $1/(4\Omega^2/\kappa^2 - 4K^2|\langle\hat{a}\rangle|^4/\kappa^2+1) \rightarrow 0$. Moreover, for a sufficiently weak anharmonicity, in a narrow frequency window enclosing the resonance and on resonance itself, we shall see next that $1/(4\Omega^2/\kappa^2 - 4K^2|\langle\hat{a}\rangle|^4/\kappa^2+1) \simeq 1$. In that case, a nonlinear broadening of the resonance is the dominant effect that stems from the presence of noise in the system. Indeed, in order to be able to approximate the value of the aforementioned distribution by 1 it is necessary that $|4\Omega^2/\kappa^2 - 4K^2|\langle\hat{a}\rangle|^4/\kappa^2| \ll 1$. The solutions to this inequality depend on the value of $K|\langle\hat{a}\rangle|^2/\kappa$. That is, the solution to the inequality is given by the sets

$$-\sqrt{\frac{K^2|\langle\hat{a}\rangle|^4}{\kappa^2}+\frac{1}{4}} \ll \frac{\Omega}{\kappa} \ll -\sqrt{\frac{K^2|\langle\hat{a}\rangle|^4}{\kappa^2}-\frac{1}{4}} \text{ and } \sqrt{\frac{K^2|\langle\hat{a}\rangle|^4}{\kappa^2}-\frac{1}{4}} \ll \frac{\Omega}{\kappa} \ll \sqrt{\frac{K^2|\langle\hat{a}\rangle|^4}{\kappa^2}+\frac{1}{4}} \text{ if } \frac{K|\langle\hat{a}\rangle|^2}{\kappa} \geq 1/2\,, \tag{53}$$

and by the set

$$-\sqrt{\frac{K^2|\langle\hat{a}\rangle|^4}{\kappa^2}+\frac{1}{4}} \ll \frac{\Omega}{\kappa} \ll \sqrt{\frac{K^2|\langle\hat{a}\rangle|^4}{\kappa^2}+\frac{1}{4}} \text{ if } 0 < \frac{K|\langle\hat{a}\rangle|^2}{\kappa} < \frac{1}{2}\,. \tag{54}$$

Using the oscillator's parameters measured in our experiment and considering either $n_{\rm th}=0$, $n_{\rm th}=0.05$ (the upper bound for the average number of thermal photons in our experiment as estimated in Supplementary Note 7) or $n_{\rm th}=1/2$, a numerical solution of the master equation (8) reveals that our anharmonicity is weak enough so as to guarantee $K|\langle\hat{a}\rangle|^2/\kappa \lesssim 0.48$ for every detuning, $-4\text{MHz} \lesssim \Delta/(2\pi) \lesssim 2.92\text{MHz}$, and all the drive powers $P_{\rm in} \leq -124$ dBm addressed in our analysis. Drive powers above $P_{\rm in}=-124$ dBm but still close to the critical power (at about $P_{\rm c} \simeq -122.2$ dBm) that sets the onset of a bistable steady-state, not only break down the condition $K|\langle\hat{a}\rangle|^2/\kappa < 1/2$, but lead to a solution that starts satisfying more narrowly the necessary assumption (27). Let us then focus on this latter regime for which $0 < K|\langle\hat{a}\rangle|^2/\kappa < 1/2$ is fulfilled. For a given driving strength resonance is attained with the detuning $\Delta_\star$ for which the magnitude $|\langle\hat{a}\rangle|$ of the steady-state amplitude reaches its maximum $|\langle\hat{a}_\star\rangle|$. Supplementary Fig. 4(a)

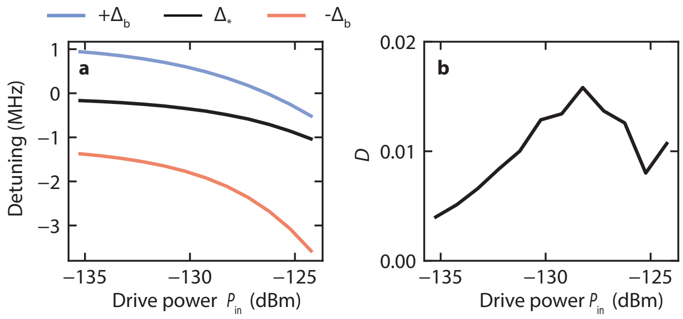

Supplementary Fig. 4. Conditions leading to a standard nonlinear broadening, proportional to $|\langle\hat{a}\rangle_\star|^2$, of the resonance of a damped driven quantum Kerr oscillator close to a Gaussian state. In a regime complying with $0 < K|\langle\hat{a}\rangle|^2/\kappa < 1/2$ for every applied input power, (**a**) the on resonance detuning, $\Delta_\star$, lies in between the detuning bounds $\pm\Delta_{\rm b}$ that derive from the frequency bounds $\pm\Omega_{\rm b}$ delimiting the on resonance equivalent of the set defined in Supp. Eq. (54) and, at the same time (**b**) both $\Omega_\star = \Delta_\star - K(2|\langle\hat{a}\rangle_\star|^2+1/2)$ and the magnitude of the oscillator's amplitude maximum $|\langle\hat{a}\rangle_\star|$ are such that the quantity $D = |[4\Omega_\star^2/\kappa^2 - 4K^2|\langle\hat{a}\rangle_\star|^4/\kappa^2+1]^{-1}-1|$ respects our approximation $D \ll 1$. All the plotted quantities are obtained from a numerical solution of the Lindblad eqation (8) with parameter values as extracted from the experimental measurements and $n_{\rm th}=1/2$.

shows the dependence on $P_{\rm in}$ of this resonance detuning $\Delta_\star$ and the detuning bounds $\pm\Delta_{\rm b} = K(2|\langle\hat{a}\rangle_\star|^2+1/2) \pm \Omega_{\rm b}$ that result from the frequency bounds $\pm\Omega_{\rm b}/\kappa = \pm\sqrt{K^2|\langle\hat{a}\rangle_\star|^4/\kappa^2+1/4}$ corresponding to the on resonance version of the set defined in Supp. Eq. (54). Note that knowledge of a maximum $|\langle\hat{a}\rangle_\star|$ as computed through a numerical solution of the master equation (8) enables us to determine both, its corresponding detuning $\Delta_\star$ as well as the ensuing detuning bounds $\pm\Delta_{\rm b}$. That is how we obtain each detuning value we plot in Supplementary Fig. 4(a), using in the numerical computation $n_{\rm th}=1/2$ and the same parameter values we extract from the experimental data. Clearly, we observe that $-\Delta_{\rm b} < \Delta_\star < \Delta_{\rm b}$, i.e., that $(-\Delta_{\rm b}, \Delta_{\rm b})$ defines an interval of detunings comprising the resonance. In Supplementary Fig. 4(b), we check the validity of the assumption $-\Delta_{\rm b} \ll \Delta_\star \ll \Delta_{\rm b}$ or, more explicitly, the relative error of the approximation $1/(4\Omega_\star^2/\kappa^2 - 4K^2|\langle\hat{a}\rangle_\star|^4/\kappa^2+1) \simeq 1$ as a function of $P_{\rm in}$, where $\Omega_\star = \Delta_\star - K(2|\langle\hat{a}\rangle_\star|^2+1/2)$. The curve shows that the deviation of the distribution from the target value 1 remains lower than a 2% for the entire range of input powers used in our theoretical analysis. The same curve as computed using either $n_{\rm th}=0$ or $n_{\rm th}=0.05$ shows an error that stays below a 5% for input powers $P_{\rm in} \leq -126$ dBm, after

which the error increases up to a 10% for $P_{\text{in}} = -124$ dBm. As we anticipated above, we may then conclude that Supp. Eq. (54) is rather well fulfilled on resonance. Supp. Eq. (54) can be equally satisfied for an arbitrarily small range of detunings $\Delta_\star - \vartheta \leq \Delta \leq \Delta_\star + \vartheta$ around the resonance, with ϑ a real constant such that $0 < \vartheta \ll |\Delta_\star|$. Thus, in order to evaluate the oscillator's steady-state amplitude near resonance, we constrain our analysis to such range of detunings and set $1/(4\Omega^2/\kappa^2 - 4K^2|\langle\hat{a}\rangle|^4/\kappa^2 + 1) \simeq 1$. This allows us to approximate the equation for the steady-state amplitude as

$$i\left[\left(\Delta - \frac{K}{2}\right)\left(1 - \frac{\gamma|\langle\hat{a}\rangle|^2}{\kappa}\right) + \frac{3K}{2} - K|\langle\hat{a}\rangle|^2 - 2K\left(n_{\text{th}} + \frac{1}{2}\right)\right]\langle\hat{a}\rangle + \frac{\kappa}{2}\left[1 + \frac{\gamma|\langle\hat{a}\rangle|^2}{\kappa}\right]\langle\hat{a}\rangle \simeq \epsilon\,. \tag{55}$$

We may now associate the resonance scenario with the approximated value of the maximum of the steady-state amplitude's magnitude that results from Supp. Eq. (55). We achieve this with the detuning $\Delta = K/2 - [3K/2 - K|\langle\hat{a}\rangle_\star|^2 - 2K(n_{\text{th}} + 1/2)][1 - \gamma|\langle\hat{a}\rangle_\star|^2/\kappa]^{-1}$ that cancels the first bracket in the left hand side of Supp. Eq. (55), such that Supp. Eq. (55) itself simplifies to $[1 + \gamma|\langle\hat{a}\rangle_\star|^2/\kappa]\langle\hat{a}\rangle_\star \simeq \alpha$, an equation that showcases the same form as the on resonance version of the steady-state equation (12) of the nonlinearly damped driven classical oscillator introduced in Supplementary Note 3. Using Cardano's method we solve the equation $[1+\gamma|\langle\hat{a}\rangle_\star|^2/\kappa]^2|\langle\hat{a}\rangle_\star|^2 \simeq \alpha^2$ for $|\langle\hat{a}\rangle_\star|^2$. The positive square root of such solution provides us with the approximated value of the maximum $|\langle\hat{a}\rangle_\star|$ that we seek, which reads

$$\begin{aligned}
|\langle\hat{a}\rangle_\star| &\simeq \left[\sqrt[3]{\frac{\alpha^2}{2\varepsilon^2} + \frac{1}{27\varepsilon^3} + \sqrt{\left(\frac{\alpha^2}{2\varepsilon^2} + \frac{1}{27\varepsilon^3}\right)^2 - \frac{1}{729\varepsilon^6}}} + \sqrt[3]{\frac{\alpha^2}{2\varepsilon^2} + \frac{1}{27\varepsilon^3} - \sqrt{\left(\frac{\alpha^2}{2\varepsilon^2} + \frac{1}{27\varepsilon^3}\right)^2 - \frac{1}{729\varepsilon^6}}} - \frac{2}{3\varepsilon}\right]^{1/2}, \\
\varepsilon &= \frac{\gamma}{\kappa} = \frac{4K^2}{\kappa^2}\left(n_{\text{th}} + \frac{1}{2}\right),
\end{aligned} \tag{56}$$

in good agreement with the data (see Supplementary Fig. 5).

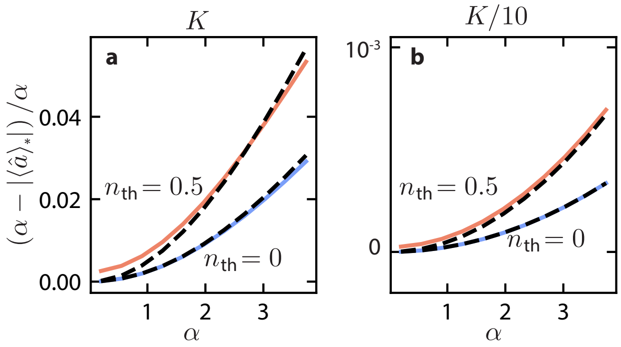

Supplementary Fig. 5. Test of the analytical model for $|\langle\hat{a}\rangle_\star|$. Numerical calculations of $|\langle\hat{a}\rangle_\star|$ (full curves) are compared to the analytical expressions of Supp. Eq. (56) (dashed curves). In panel **a** we use the same regime of parameters as the measured device, notably the same Kerr parameter K, with both no thermal population $n_{\text{th}} = 0$ and half a quantum of thermal population $n_{\text{th}} = 1/2$. In panel **b**, we show that the accuracy of the analytical model increases as we decrease K by a factor 10. For the calculations with thermal population, good agreement is found in the regime $n_{\text{th}} \ll |\langle\hat{a}\rangle|^2$ covered by our assumptions.

Similarly to Supplementary Note 3, to ease the subsequent analysis and acquire a clearer insight of the behaviour of the oscillator's resonant amplitude, we derive next a simpler approximated solution to Supp. Eq. (55) based on perturbation theory. Given that $K|\langle\hat{a}\rangle|^2/\kappa < 1/2$, if we further take into account our original assumptions of weak anharmonicity and strength of thermal fluctuations, c. f. (25) and (24), respectively, we find that $\varepsilon \ll 1$. The solution of the oscillator's steady-state amplitude that we seek comes from a series expansion $\langle\hat{a}\rangle = \sum_{l=0}\varepsilon^l\langle\hat{a}\rangle_l$ in powers of the small parameter $\varepsilon = \gamma/\kappa$. For our simple description of the resonance, we content ourselves with the regime for which the first order correction in ε is dominant. This leaves us with the expansions $\langle\hat{a}\rangle \simeq \langle\hat{a}\rangle_0 + \varepsilon\langle\hat{a}\rangle_1$ and $|\langle\hat{a}\rangle|^2 \simeq |\langle\hat{a}\rangle_0|^2 + \varepsilon[\langle\hat{a}\rangle_0^*\langle\hat{a}\rangle_1 + \langle\hat{a}\rangle_1^*\langle\hat{a}\rangle_0]$. Likewise, we expand the detuning in the form $\Delta = \Delta_0 + \varepsilon\Delta_1$. By replacing these expansions into Supp. Eq. (55), neglecting terms of order higher than ε^1 and grouping terms of the same order

in ε, we obtain the following set of equations

$$i\left[\Delta_0 + K - K|\langle\hat{a}\rangle_0|^2 - 2K\left(n_{\text{th}} + \frac{1}{2}\right)\right]\langle\hat{a}\rangle_0 + \frac{\kappa}{2}\langle\hat{a}\rangle_0 = \epsilon\,, \tag{57}$$

$$i\left[\Delta_1 - |\langle\hat{a}\rangle_0|^2\Delta_0 + \frac{K}{2}|\langle\hat{a}\rangle_0|^2 - K(\langle\hat{a}\rangle_0^*\langle\hat{a}\rangle_1 + \langle\hat{a}\rangle_1^*\langle\hat{a}\rangle_0)\right]\langle\hat{a}\rangle_0$$
$$+\,i\left[\Delta_0 + K - K|\langle\hat{a}\rangle_0|^2 - 2K\left(n_{\text{th}} + \frac{1}{2}\right)\right]\langle\hat{a}\rangle_1 + \frac{\kappa}{2}\left[\langle\hat{a}\rangle_1 + |\langle\hat{a}\rangle_0|^2\right] \simeq 0\,. \tag{58}$$

In the resonance scenario described by the set of equations above, the detuning must be such that it cancels each of the brackets multiplied by the imaginary unit in the left hand side of Supp. Eqs. (57) and (58). Solving the resulting equations for the corresponding resonant amplitude yields $\langle\hat{a}\rangle_\star \simeq \langle\hat{a}\rangle_{\star,0} + \varepsilon\langle\hat{a}\rangle_{\star,1}$ with $\langle\hat{a}\rangle_{\star,0} = \alpha$ and $\langle\hat{a}\rangle_{\star,1} \simeq -|\langle\hat{a}\rangle_0|^2\langle\hat{a}\rangle_0 = -\alpha^3$. The simple approximation for the maximum of the absolute value of the amplitude is maximized for

$$\Delta = \Delta_{\star,0} + \frac{4K^2\alpha^2}{\kappa^2}\left(n_{\text{th}} + \frac{1}{2}\right)(\Delta_{\star,0} - 2K\alpha^2 - K/2)\,, \tag{59}$$

where $\Delta_{\star,0} = -K + K\alpha^2 + 2K(n_{\text{th}} + 1/2)$, and reads

$$|\langle\hat{a}\rangle_\star| \simeq \alpha\left(1 - \frac{4K^2\alpha^2}{\kappa^2}\left(n_{\text{th}} + \frac{1}{2}\right)\right)\,. \tag{60}$$

There are two corrections to the unperturbed amplitude α: one due to the quantum commutation relations, and the other are due to thermal effects. We note that other authors have already derived more precise analytical equations for the amplitude (in absence of thermal noise) [14, 17, 18].

G. Discussion

The fact that an inclusion of noise leads to the same equations as the nonlinear damping model is confirmed by our fit of the latter model to data. As covered in Supplementary Note 3, the nonlinear damping is found to be $2\pi \times 5.02$ kHz, which is close to the value expected from this calculation. Indeed, in absence of thermal noise $n_{\text{th}} = 0$, Supp. Eq. (52) gives $\gamma = 2K^2/\kappa = 2\pi \times 5.6$ kHz.

We can also use the approximate description of the resonance scenario embodied in Supp. Eqs. (59) and (60) above to provide a value for the corresponding average photon number. Given its equation of motion (40), in the steady-state and on resonance, we have $\langle\hat{a}^\dagger\hat{a}\rangle_\star = n_{\text{th}} + \alpha(\langle\hat{a}\rangle_\star + \langle\hat{a^\dagger}\rangle_\star)/2$. Based on our previous perturbation series expansion $\langle\hat{a}\rangle_\star \simeq \alpha[1 - \gamma\alpha^2/\kappa]$, and hence

$$\langle\hat{a}^\dagger\hat{a}\rangle_\star \simeq n_{\text{th}} + \alpha^2\left(1 - \frac{4K^2\alpha^2}{\kappa^2}\left(n_{\text{th}} + \frac{1}{2}\right)\right) = \alpha^2\left(1 + \frac{n_{\text{th}}}{\alpha^2} - \frac{4K^2\alpha^2}{\kappa^2}\left(n_{\text{th}} + \frac{1}{2}\right)\right)\,, \tag{61}$$

yielding, in the limit given by our assumptions (25) and (24), the following result

$$\sqrt{\langle\hat{a}^\dagger\hat{a}\rangle_\star} \simeq \alpha\left(1 + \frac{n_{\text{th}}}{2\alpha^2} - \frac{2K^2\alpha^2}{\kappa^2}\left(n_{\text{th}} + \frac{1}{2}\right)\right)\,. \tag{62}$$

The interplay of noise and Kerr nonlinearity leads to a reduction in $\sqrt{\langle\hat{a}^\dagger\hat{a}\rangle_\star}$ equal to half the reduction in $|\langle\hat{a}\rangle_\star|$ (Supp. Eq. (62)). The steady-state of the driven-dissipative Kerr system differs thus from a coherent state, for which $\sqrt{\langle\hat{a}^\dagger\hat{a}\rangle_\star} = |\langle\hat{a}\rangle_\star|$, or a thermal coherent state, for which $\sqrt{\langle\hat{a}^\dagger\hat{a}\rangle_\star} \simeq |\langle\hat{a}\rangle_\star| + n_{\text{th}}/(2|\langle\hat{a}\rangle_\star|)$ if $n_{\text{th}} \ll |\langle\hat{a}\rangle_\star|$. We can thus attribute half of the reduction of amplitude $|\langle\hat{a}\rangle_\star|$ to a reduction of photon number or energy of the oscillator.

The subscript $\star$ is omitted in the rest of the manuscript where the resonant condition is implied through context.

SUPPLEMENTARY NOTE 5 – WIGNER CURRENT

A. Mathematical details

We follow Ref. [19] to write the evolution of the Wigner function W as a phase-space continuity equation

$$\partial_t W + \boldsymbol{\nabla} \boldsymbol{J} = 0 \ , \tag{63}$$

where $\nabla = \begin{pmatrix} \partial_x \\ \partial_p \end{pmatrix}$ and $\boldsymbol{J} = \begin{pmatrix} J_x \\ J_p \end{pmatrix}$ denotes the Wigner current.

This method has already been used to study the Kerr oscillator, however in the absence of a driving force [20, 21] or in a non-rotating frame [22]. The slightly different Hamiltonian of the Duffing oscillator, this time with both a driving force and damping has also been studied, however not in the rotating frame [23]. Here we derive an expression for the Wigner current of the driven-dissipative Kerr oscillator in the rotating frame, characterized by the Hamiltonian

$$\hat{H}/\hbar = \left(\Delta + \frac{K}{2}\right) \hat{a}^\dagger \hat{a} - \frac{K}{2} \left(\hat{a}^\dagger \hat{a}\right)^2 + i\epsilon(\hat{a}^\dagger - \hat{a}) \ . \tag{64}$$

The slight difference in the form of nonlinearity with respect to Supp. Eq. (1) (allowed by the commutation relations) makes for a more favorable expression when Wigner-transforming the Hamiltonian to phase space coordinates x, p. We introduce the phase-space operators as $\hat{x}, \hat{p}$ through

$$\hat{a} = \frac{1}{\sqrt{2}} \left(\hat{x} + i\hat{p}\right) \ , \tag{65}$$

such that $[\hat{x}, \hat{p}] = i$, yielding

$$\hat{H}/\hbar = \frac{1}{2} \left(\Delta + K\right) \left(\hat{x}^2 + \hat{p}^2\right) - \frac{K}{8} \left(\hat{x}^2 + \hat{p}^2\right)^2 + \epsilon\sqrt{2}\hat{p} \ , \tag{66}$$

omitting constant contributions. To compute the Wigner current we first need the Wigner transform of the Hamiltonian, also known as the inverse of the Weyl transform, defined by [19]

$$H(x,p) = 2 \int_{-\infty}^{+\infty} \mathrm{d}z e^{\frac{2ipz}{\hbar}} \left\langle x - z|\hat{H}|x + z \right\rangle \ , \tag{67}$$

which yields

$$H(x,p)/\hbar = \frac{1}{2} \left(\Delta + K\right) \left(x^2 + p^2\right) - \frac{K}{8} \left(x^2 + p^2\right)^2 + \epsilon\sqrt{2}p \ , \tag{68}$$

omitting constant contributions. It is easier to prove that $\hat{H}$ is the Weyl transform of $H(x,p)$ rather than the fact that $H(x,p)$ is the Wigner transform of $\hat{H}$. To do so, we may use the McCoy formula [24]

$$p^m x^n \quad \longmapsto \quad \frac{1}{2^n} \sum_{r=0}^{n} \binom{n}{r} \hat{x}^r \hat{p}^m \hat{x}^{n-r} \ , \tag{69}$$

where $\longmapsto$ designates a Weyl transformation. A consequence of this formula is that

$$\begin{aligned} x^n &\quad \longmapsto \quad \hat{x}^n \\ p^n &\quad \longmapsto \quad \hat{p}^n \\ 2p^2x^2 &\quad \longmapsto \quad \frac{1}{2}\left(\hat{p}^2\hat{x}^2 + 2\hat{x}\hat{p}^2\hat{x} + \hat{x}^2\hat{p}^2\right) \\ &\qquad\quad = \hat{x}^2\hat{p}^2 + \hat{p}^2\hat{x}^2 + 1 \end{aligned} \tag{70}$$

The two first relations prove the correspondence between the harmonic and driving terms. Utilizing all three relations, we can demonstrate the correspondance between the Kerr terms in $\hat{H}$ and $H(x,p)$, up to a constant factor which plays no role in successive manipulations of $H(x,p)$.

The unitary evolution of the Wigner function, equivalent to the evolution of the state vector dictated by Schrödinger's equation, is given by [19]

$$\partial_t W(x,p,t) = \{\{H,W\}\} \equiv \frac{2}{\hbar}\, H(x,p)\, \sin\left(\frac{1}{2}\left(\overleftarrow{\partial}_x\overrightarrow{\partial}_p - \overleftarrow{\partial}_p\overrightarrow{\partial}_x\right)\right)\, W(x,p,t), \tag{71}$$

where $\{\{H,W\}\}$ is called the Moyal bracket. The arrows above the partial derivatives indicates whether the term on the right or left should be differentiated. For example:

$$H(x,p)\, \overleftarrow{\partial}_x\, W(x,p,t) = (\partial_x H(x,p))\, W(x,p,t) \tag{72}$$

and

$$H(x,p)\, \overrightarrow{\partial}_x\, W(x,p,t) = H(x,p)\, (\partial_x W(x,p,t)) \ . \tag{73}$$

Since the Hamiltonian only contains terms $x^n p^m$ with $n+m \leq 4$, we may write in this context

$$\sin\left(\frac{1}{2}(\overleftarrow{\partial}_x\overrightarrow{\partial}_p - \overleftarrow{\partial}_p\overrightarrow{\partial}_x)\right) = \frac{1}{2}(\overleftarrow{\partial}_x\overrightarrow{\partial}_p - \overleftarrow{\partial}_p\overrightarrow{\partial}_x) - \frac{1}{3!}\frac{1}{2^3}(\overleftarrow{\partial}_x\overrightarrow{\partial}_p - \overleftarrow{\partial}_p\overrightarrow{\partial}_x)^3 \ . \tag{74}$$

The Moyal bracket for the harmonic part of the Hamiltonian writes

$$\begin{aligned} &2\,\frac{1}{2}\,(\Delta+K)\left(x^2+p^2\right)\frac{1}{2}(\overleftarrow{\partial}_x\overrightarrow{\partial}_p - \overleftarrow{\partial}_p\overrightarrow{\partial}_x)W(x,p,t) \\ &= (\Delta+K)\,(x\,\overrightarrow{\partial}_p - p\,\overrightarrow{\partial}_x)W(x,p,t) \\ &= -\begin{pmatrix}\partial_x \\ \partial_p\end{pmatrix} \underbrace{\begin{pmatrix} p \\ -x\end{pmatrix}(\Delta+K)\,W(x,p,t)}_{\boldsymbol{J}_{\text{harmonic}}} \ . \end{aligned} \tag{75}$$

For the drive term

$$2\epsilon\sqrt{2}p\frac{1}{2}(\overleftarrow{\partial}_x\overrightarrow{\partial}_p - \overleftarrow{\partial}_p\overrightarrow{\partial}_x)W(x,p,t) \tag{76}$$

$$= -\,\epsilon\sqrt{2}\,\overrightarrow{\partial}_x\, W(x,p,t) \tag{77}$$

$$= -\begin{pmatrix}\partial_x \\ \partial_p\end{pmatrix} \underbrace{\begin{pmatrix} 1 \\ 0\end{pmatrix}\epsilon\sqrt{2}W(x,p,t)}_{\boldsymbol{J}_{\text{drive}}} \ . \tag{78}$$

The first order derivatives of the Moyal bracket applied to the nonlinearity write

$$\begin{aligned} &-2\frac{K}{8}\left(x^2+p^2\right)^2\frac{1}{2}(\overleftarrow{\partial}_x\overrightarrow{\partial}_p - \overleftarrow{\partial}_p\overrightarrow{\partial}_x)W(x,p,t) \\ &= -\frac{K}{2}\left(x^2+p^2\right)(x\,\overrightarrow{\partial}_p - p\,\overrightarrow{\partial}_x)W(x,p,t) \\ &= -\begin{pmatrix}\partial_x \\ \partial_p\end{pmatrix} \underbrace{\begin{pmatrix} p \\ -x\end{pmatrix}\left(-\frac{K}{2}\left(x^2+p^2\right)\right)W(x,p,t)}_{\boldsymbol{J}_{\text{Kerr},1}} \ . \end{aligned} \tag{79}$$

And the higher order derivatives of the Moyal bracket applied to the nonlinearity yield

$$\begin{aligned} &-2\frac{K}{2}\frac{1}{4}\left(x^2+p^2\right)^2\left(-\frac{1}{3!}\frac{1}{2^3}(\overleftarrow{\partial}_x\overrightarrow{\partial}_p - \overleftarrow{\partial}_p\overrightarrow{\partial}_x)^3\right)W(x,p,t) \\ &= -\begin{pmatrix}\partial_x \\ \partial_p\end{pmatrix} \underbrace{\frac{K}{24}\begin{pmatrix} p(3\partial_{xx}+\partial_{pp}) - x(\partial_{xp}+\partial_{px}) \\ -x(\partial_{xx}+3\partial_{pp}) + p(\partial_{xp}+\partial_{px})\end{pmatrix}W(x,p,t)}_{\boldsymbol{J}_{\text{Kerr},2}} \ , \end{aligned} \tag{80}$$

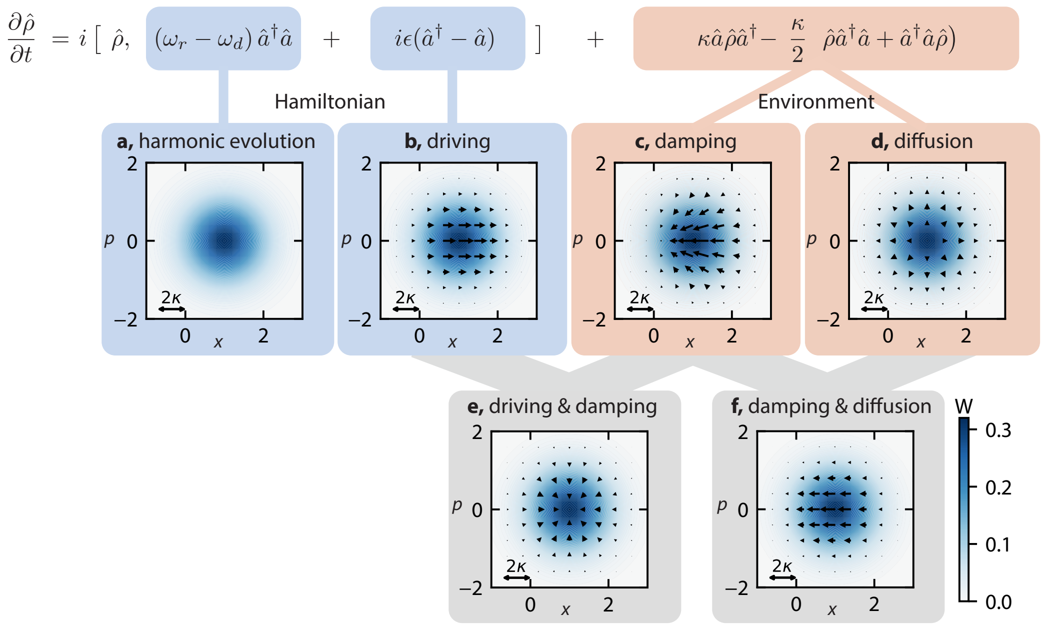

Supplementary Fig. 6. **Wigner current in a resonantly-driven harmonic oscillator.** We overlay the Wigner function of the steady-state (a coherent state), with the Wigner current associated with the different terms of the Lindblad equation. The sum of these vector fields $\boldsymbol{J}$ plays the role of a current in a continuity equation $\partial W/\partial t = \boldsymbol{\nabla J}$ equivalent to the Lindblad equation. **a**, On resonance $\omega_\mathrm{r} = \omega_\mathrm{d}$, there is no harmonic evolution in the rotating frame. **b**, The driving moves the Wigner function towards increasing x. **c**, The damping moves the Wigner function towards the origin. **d**, The diffusion term (quantum noise) expands the Wigner function in all directions. **e**, Since the drive acts along the $x-$axis and the damping acts more radially, these two terms do not counteract each other. Rather, they move the Wigner function towards a point in phase space, the classical solution to the problem. This is counteracted by the diffusion (quantum noise). Together, these effects give the coherent state its finite size in phase space. **f**, Another way of showing this is to add the effect of the damping and diffusion, which yields a current acting in the x-axis rather than radially, which counteracts the driving.

which in our regime of parameters has a negligibly small contribution to the total current. The non-unitary evolution of the Wigner function, equivalent to the Lindblad equation of Supp. Eq. (8), is given by [23]

$$\partial_t W = \{\{H, W\}\} \ - \begin{pmatrix}\partial_x\\ \partial_p\end{pmatrix} \left(\underbrace{-\frac{\kappa}{2}\begin{pmatrix}x\\ p\end{pmatrix} W}_{\boldsymbol{J}_\mathrm{damping}} \underbrace{-\frac{\kappa}{2}\left(n_\mathrm{th}+\frac{1}{2}\right)\begin{pmatrix}\partial_x\\ \partial_p\end{pmatrix} W}_{\boldsymbol{J}_\mathrm{diffusion}} \right) . \tag{81}$$

These expressions are utilized in the discussion surrounding Fig. 4. We supplement Fig. 4 by the discussion below, where we provide details on the construction of the figure and further arguments in favor of its interpretation. We also provide in Supplementary Fig. 8 a detailed plot of the Wigner currents for the steady-state shown in Fig. 4**c**. Finally, we plot in Supplementary Fig. 6 and Supplementary Fig. 7 the Wigner currents for the pedagogical cases of a resonantly and off-resonantly driven harmonic oscillators respectively.

B. Supplementary information for Fig. 4

In Fig. 4**b**, we show a coherent state, of amplitude α, and the state which results from evolving that coherent state under the Kerr effect $\hat H/\hbar = \lambda K\hat a^\dagger\hat a - (K/2)\hat a^\dagger\hat a^\dagger\hat a\hat a$ for a time $t = 1/(45K)$. This unitary evolution is computed using QuTiP [25, 26]. To ensure that the state is centered around the x axis in phase space, we add a harmonic evolution characterized by the coefficient $\lambda = 15$. After evolution of the coherent state due to the Kerr effect, the average photon number remains unchanged. This is theoretically expected since the Hamiltonian dictating this evolution $\hat H = \hbar\lambda K\hat a^\dagger\hat a - \hbar(K/2)\hat a^\dagger\hat a^\dagger\hat a\hat a$ commutes with the photon number operator $\hat a^\dagger\hat a$.

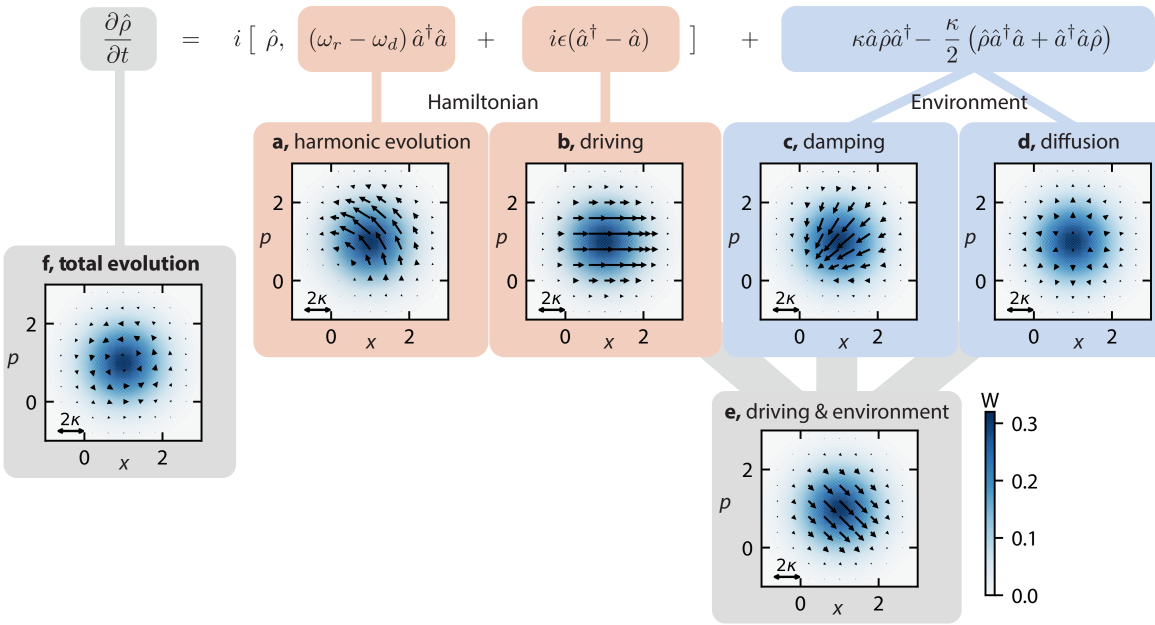

Supplementary Fig. 7. Wigner current in an off-resonantly driven harmonic oscillator. We overlay the harmonic **a**, drive **b**, damping **c**, and diffusion (quantum noise) **d** Wigner currents on the Wigner distribution of the steady-state. **e**, Contrary to the resonant case, the sum of diffusion and damping no longer cancel the drive. Instead, these currents point away from the direction of the harmonic evolution, such that the net total current is 0. **f**, The remaining current moves points of the Wigner distribution along lines of equal probability such that $\partial W/\partial t = 0$.

The amplitude $|\langle\hat{a}\rangle|$, or the distance between the center of mass of the distribution and the origin in phase space, decreases. In Fig. 4**b**, the decrease in amplitude is of 0.34 percent, which translates to a considerable 0.33 dB difference in $|S_{21}|$. We have called this effect A.

As a result of the deformation of the Wigner distribution, the drive is less affective at countering the environmental effects (noise and damping). If we call $\boldsymbol{J}_{\text{env}} = \boldsymbol{J}_{\text{damping}} + \boldsymbol{J}_{\text{diffusion}}$, and integrate the absolute value of this current, and the driving current over one of the distribution of Fig. 4**b**, we find $\iint|\boldsymbol{J}_{\text{env}}|\mathrm{d}x\mathrm{d}p = \iint|\boldsymbol{J}_{\text{drive}}|\mathrm{d}x\mathrm{d}p$. Whilst this is true for the coherent state and the state deformed by the Kerr effect, we obtain a different result if we project the driving current in the same direction as the environmental effects. For a coherent state, we have $\iint|\boldsymbol{J}_{\text{env}}|\mathrm{d}x\mathrm{d}p = \iint|\boldsymbol{J}_{\text{drive}}\cdot\frac{\boldsymbol{J}_{\text{env}}}{|\boldsymbol{J}_{\text{env}}|}|\mathrm{d}x\mathrm{d}p$. So the drive is aligned with the environmental effects and exactly counters them. However, for the deformed state $\iint|\boldsymbol{J}_{\text{env}}|\mathrm{d}x\mathrm{d}p = 6.10$ MHz, and $\iint|\boldsymbol{J}_{\text{drive}}\cdot\frac{\boldsymbol{J}_{\text{env}}}{|\boldsymbol{J}_{\text{env}}|}|\mathrm{d}x\mathrm{d}p = 6.07$ MHz. So after effect of the Kerr, whilst the drive matches the environmental effects in absolute magnitude, in the direction of the environmental current the driving current is smaller. The Wigner distribution thus tends to move towards the origin in phase space, hence reducing the total photon number. We have called this effect B.

This is further illustrated if we track the evolution of various observables as a coherent state of amplitude α evolves to the steady-state following Supp. Eq. (8), see Supplementary Fig. 9. We look at the amplitude $|\langle\hat{a}\rangle| = |\mathrm{Tr}(\hat{a}\hat{\rho})|$, the square root of the number of photons $\sqrt{\langle\hat{a}^\dagger\hat{a}\rangle} = \sqrt{\mathrm{Tr}(\hat{a}^\dagger\hat{a}\ \hat{\rho})}$ and the variance of the phase $\Delta\varphi = \sqrt{\mathrm{Tr}(\hat{\varphi}^2\hat{\rho}) - \mathrm{Tr}(\hat{\varphi}\hat{\rho})^2}$. Additionally, we look at the time derivative of these observables induced by various terms of Supp. Eq. (8).

We distinguish the influence of different terms by computing the change in density matrix induced by drive and damping

$$\left[\frac{\partial\hat{\rho}}{\partial t}\right]_{\kappa,\epsilon} = -i\left[i\epsilon(\hat{a}^\dagger - \hat{a}), \hat{\rho}\right] + \kappa(n_{\text{th}}+1)D(\hat{a})\hat{\rho} + \kappa n_{\text{th}}D(\hat{a}^\dagger)\hat{\rho}\ , \tag{82}$$

and by the harmonic and Kerr evolution

$$\left[\frac{\partial\hat{\rho}}{\partial t}\right]_{K} = -i\left[\Delta\hat{a}^\dagger\hat{a} - \frac{K}{2}\hat{a}^\dagger\hat{a}^\dagger\hat{a}\hat{a}, \hat{\rho}\right]\ . \tag{83}$$

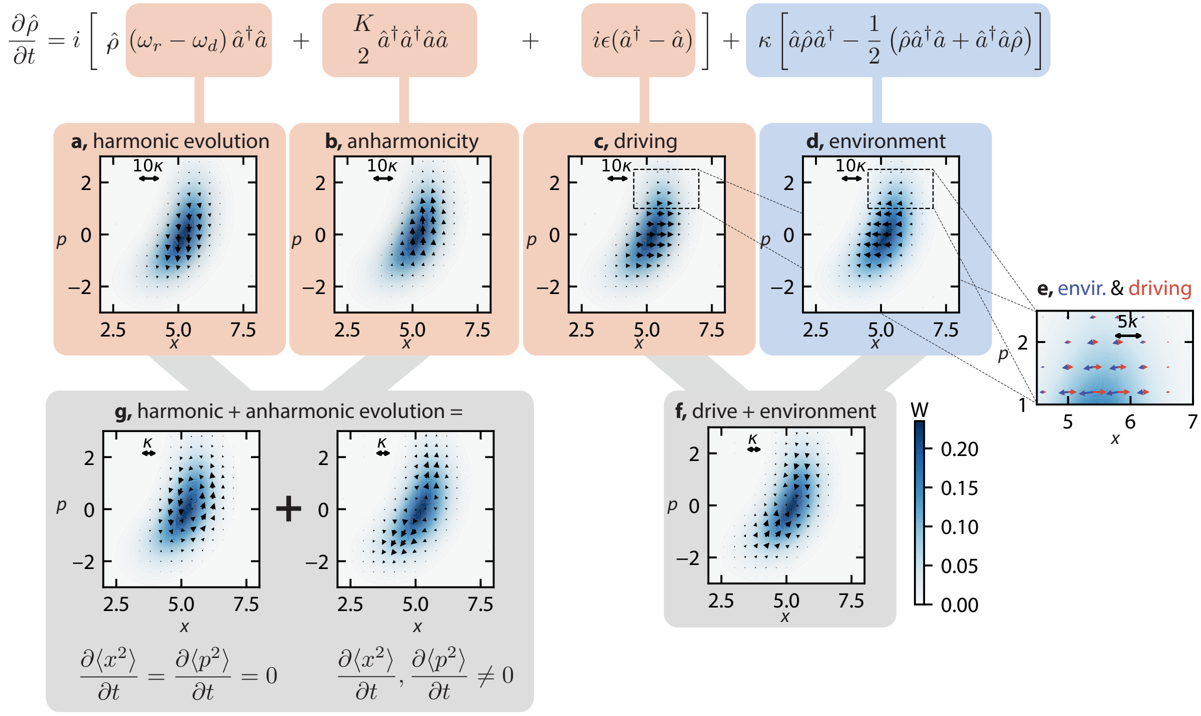

Supplementary Fig. 8. Wigner current in the steady-state of the driven Kerr oscillator. We overlay the contributions of the harmonic **a**, anharmonic **b**, drive **c**, and environmental **d** terms of the Lindblad equation on the Wigner function of Fig. 4**e**. **e**, Zoom-in showing both environmental and driving contributions. Whilst the drive acts on the $x-$axis, the environment acts more radially. They only partially compensate each other, and the result of summing these currents is shown in **f**. Together, they tend to diminish the spread in phase of the Wigner function. **g**, This is compensated by the anharmonicity which tends to increase the spread in phase. To make this clear, we have divided the sum of the harmonic and anharmonic currents into two contributions. In **g**-left we have shown the part of the current which preserves the spread in position $\partial\langle x^2\rangle/\partial t = \partial\langle p^2\rangle/\partial t = 0$. This corresponds to the total Wigner current. In **g**-right we have shown the part of the current which increases the spread in position, and exactly compensates the combined effect of the drive and environment. The latter is plotted in Fig. 4**c**.

Given a change in density matrix $\frac{\partial\hat\rho}{\partial t}$, we can compute the change in amplitude from

$$\begin{aligned}\frac{\partial|\langle\hat a\rangle|}{\partial t} &= \frac{\partial}{\partial t}\sqrt{\langle\hat a\rangle^*\langle\hat a\rangle}\\ &= \frac{1}{2}\left(\left(\frac{\partial}{\partial t}\langle\hat a\rangle\right)^*\langle\hat a\rangle + \langle\hat a\rangle^*\left(\frac{\partial}{\partial t}\langle\hat a\rangle\right)\right)/|\langle\hat a\rangle|\ ,\end{aligned} \tag{84}$$

where

$$\begin{aligned}\frac{\partial}{\partial t}\langle\hat a\rangle &= \frac{\partial}{\partial t}\mathrm{Tr}(\hat a\hat\rho)\\ &= \mathrm{Tr}(\hat a\frac{\partial\hat\rho}{\partial t})\ .\end{aligned} \tag{85}$$

For the photon number

$$\frac{\partial}{\partial t}\langle\hat a^\dagger\hat a\rangle = \mathrm{Tr}(\hat a^\dagger\hat a\frac{\partial\hat\rho}{\partial t})\ . \tag{86}$$

And for the variance in the phase

$$\frac{\partial}{\partial t}\Delta\varphi = \frac{\mathrm{Tr}(\hat\varphi^2\frac{\partial\hat\rho}{\partial t}) - 2\mathrm{Tr}(\hat\varphi\hat\rho)\mathrm{Tr}(\hat\varphi\frac{\partial\hat\rho}{\partial t})}{2\Delta\varphi}\ . \tag{87}$$

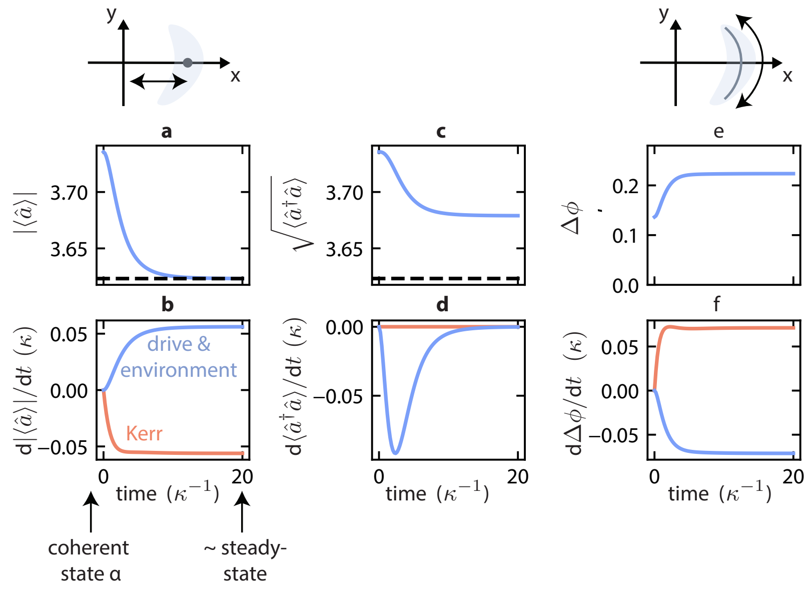

Supplementary Fig. 9. **Evolution of the driven-dissipative Kerr oscillator starting in a coherent state.** We compute the time-evolution of the density matrix following Supp. Eq. (8) in QuTiP [25, 26]. The starting state is taken to be a coherent state of amplitude α (see Fig. 4**a**), the amplitude corresponding to the classical solution to the problem. The simulation is carried out with a power $P_{\text{in}} = -124$, until the steady-state shown in Fig. 4**c** is reached. The time evolution of various observables are plotted over time in order to further illustrate the amplitude reduction mechanism shown in Fig. 4. We see in **b** that the Kerr causes $|\langle\hat{a}\rangle|$ to reduce over time (negative time-derivative) whereas the drive and environment counteracts this effect (positive time-derivative) until equilibrium is reached at the steady-state (the total time-derivative is 0). This causes the reduction of $|\langle\hat{a}\rangle|$ shown in **a**. The dashed line corresponds to the steady-state value for $|\langle\hat{a}\rangle|$ in **a** and **c**. In **c**, we plot the square of the average photon number, and see that a reduction in photon number accounts for half of the total reduction in $|\langle\hat{a}\rangle|$. From **d**, we find that the photon number is conserved under the Kerr evolution (derivative is 0), whereas the interaction of the drive and environment is at the origin of the decrease. The phase-space interpretation of this effect is that through an increase in the phase variance, the environmental Wigner current is no longer parallel to the driving current (see Fig. 4**c**), creating a net current of the probability towards the origin (i.e. a reduction in photon number). Since the amplitude of the damping current is reduced closer the origin (*i.e.* is smaller for lower photon numbers), whereas the drive is not, equilibrium is found when the probability gets closer to the origin. In **e**, we plot the evolution of the variance in phase $\Delta\phi$. Its increase is shown to be due to the Kerr effect in **f**, and countered by the interaction of drive and environment.

Consistently with the explanations surrounding Fig.4, we observe that the Kerr effect causes a decrease of the amplitude (Supplementary Fig. 9**b**) (effect A). And this effect is eventually compensated by the combined effect of the drive and environment. We also observe that the average number of photons decreases due to the interaction of drive and damping (Supplementary Fig. 9**f**), and this effect seems to follow the increase in phase variance (Supplementary Fig. 9**f**) (effect B).

Whilst the explanations above and surrounding Fig. 4 help in understanding the physical mechanism behind the dephasing which manifests in the same way as damping, they do not elucidate its nonlinear nature. To understand the latter, we turn to estimating the equilibrium of Wigner currents (see Supplementary Fig. 10 for a visual point of reference). We consider the state of Fig. 4**b**, after the Kerr effect has acted on the coherent state. Assuming we are driving at the new resonance frequency[27] $\Delta = K\alpha^2$, we determine the scaling of currents for a point which has undergone a characteristic amount of dephasing $\Delta\varphi$. We consider a point in phase space which has a distance from the origin given by $\langle\hat{x}\rangle + \Delta x$, where $\Delta x = 1/\sqrt{2}$ is the uncertainty in position of a coherent state and $\langle\hat{x}\rangle = \epsilon/(\sqrt{2}\kappa)$. We find that the Kerr and harmonic evolution Wigner current $\boldsymbol{J}_{\text{Kerr},1} + \boldsymbol{J}_{\text{harmonic}}$ scales with ϵ^2, and points in a perpendicular direction to the damping. Our numerics show that in practice the second contribution to the Kerr current $\boldsymbol{J}_{\text{Kerr},2}$ is negligibly small, and we see in Fig. 4**c** that the influence of quantum noise at large dephasing is also negligible. In this discussion we will simplify the steady-state condition as requiring the orthogonal Wigner currents of Kerr/harmonic evolution and damping to be matched by the drive which is oriented horizontally. As illustrated in Supplementary Fig. 10 this condition leads to an understanding of the nonlinear evolution of the apparent damping, which decreases the total amplitude α by a factor ϵ^2, resulting in the nonlinear scaling of amplitude $\alpha\epsilon^2 \propto \alpha^3$ found both experimentally and theoretically.

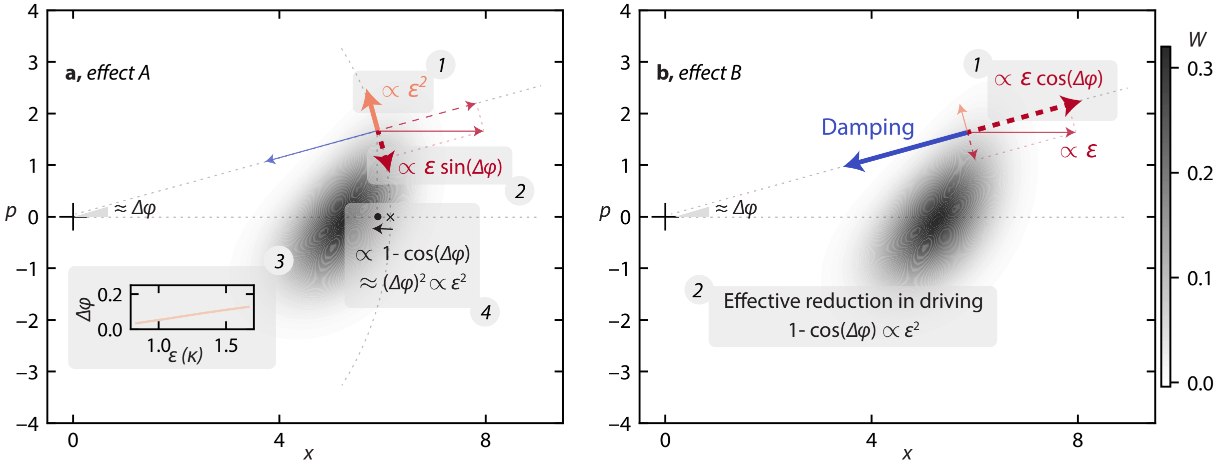

Supplementary Fig. 10. **Origin of the nonlinear nature of the damping.** Following a unitary evolution of a coherent state due to the Kerr effect, the state acquires an uncertainty in phase characterized by $\Delta\varphi$. We show in both panels the Wigner function of Fig. 4**b** illustrating this effect. The Wigner currents are drawn with arbitrary lengths for pedagogical purposes and do not reflect their true values. **(a -1)** A point in phase space which has acquired a phase offset $\Delta\varphi$ will be subject to the Kerr and harmonic evolution scaling with the amplitude squared, or in other words ϵ^2. (2) When the damping and driving are active, the steady-state is reached when the Kerr current is compensated by the tangential component of the drive which scales with $\epsilon \sin(\Delta\varphi)$. (3) For an equilibrium to occur, and assuming the dephasing is small $\sin(\Delta\varphi) \simeq \Delta\varphi$, the dephasing should scale linearly with ϵ. In the inset we show a simulation of the phase uncertainty $\Delta\varphi = \Delta\phi - 1/n$ which is acquired with increasing driving. To compute $\Delta\varphi$, we have subtracted the phase uncertainty of a coherent state $1/n = 1/\langle\hat{a}^\dagger\hat{a}\rangle$, which does not contribute to the amplitude damping, to the total uncertainty in phase $\Delta\phi$. As expected, $\Delta\varphi$ increases linearly with ϵ. (4) By projecting the equilibrium point on the axis of the center of mass of the distribution (COM), we find a contribution to the reduction of the COM proportional to ϵ^2 . This scaling is consistent with our experimental and theoretical findings, and helps to understand why effect A leads to an apparent damping which is nonlinear in ϵ. **b** When we look at the balance of currents in the radial direction, we find that the phase offset $\Delta\varphi$ causes the contribution of the driving current to be reduced by a factor $(\epsilon - \epsilon\cos(\Delta\varphi))/\epsilon \simeq \epsilon^2$. This is consistent with the reduction in photon number proportional to the amplitude squared, and helps to understand why effect B leads to an apparent nonlinear damping.

SUPPLEMENTARY NOTE 6 – QUADRATURE SQUEEZING

We determine the degree of squeezing of the steady-state solutions to Supp. Eq. (8) for different powers. We consider squeezing along the orthonormal coordinate set u, v with $u = \cos(\theta)x + \sin(\theta)p$. The coordinate u corresponds to the operator $\hat{u} = e^{i\theta}\hat{a}^\dagger + e^{-i\theta}\hat{a}$ for which we compute the uncertainty Δu for varying θ. As shown in Supplementary Fig. 11, for each measured power, there is a angle θ for which the uncertainty is below the uncertainty of a coherent state, demonstrating quadrature squeezing.

SUPPLEMENTARY NOTE 7 – EXPERIMENTAL SETUP

A. Experimental setup

The edges of the chip are wire-bonded to a printed circuit board (PCB) and the chip is placed in a copper box thermally anchored to the 20 milliKelvin stage of a dilution refrigerator. The input to the device is wired through the PCB to the output of a room-temperature vector network analyzer (VNA), the signal coming from the VNA is attenuated at each plate of the dilution refrigerator. The cryogenic wiring is detailed in Supplementary Fig. 12.

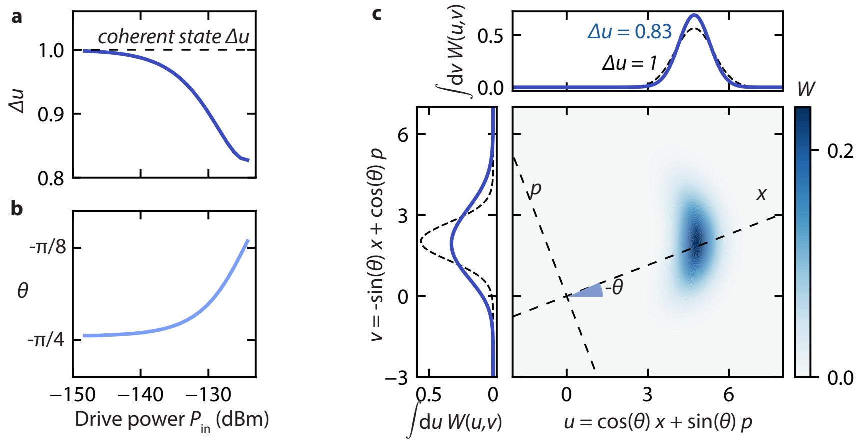

Supplementary Fig. 11. Quadrature squeezing. The angle θ defines the orthonormal coordinate set u, v with $u = \cos(\theta)x + \sin(\theta)p$ where the uncertainty in $\hat{u}$ named Δu is minimized. For each driving power, we plot the minimum Δu in **a** and the angle which achieves this minimum in **b**. The uncertainty Δu is expressed relative to the quadrature uncertainty of a coherent state shown as a dashed line. **c**, Wigner function plotted in the u, v basis for $P_{\text{in}} = -124.2$ dBm where the smallest Δu is achieved. On the left and on top, we plot the marginal distributions for u and v respectively (blue), overlayed with the distributions of a coherent state (dashed lines) with the same amplitude $\langle \hat{a} \rangle$ as the plotted Wigner function. In this case, the uncertainty Δu for the steady-state is 83 percent of the uncertainty one would obtain for a coherent state, demonstrating quadrature squeezing.

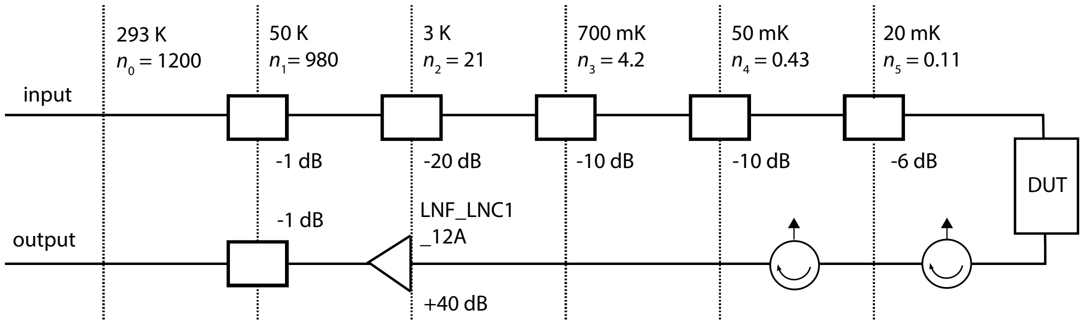

Supplementary Fig. 12. Cryogenic wiring diagram. On the input side, at each temperature stage (corresponding to a plate of our dilution refrigerator) the signal passes through a thermalized attenuator. This reduces the noise photon occupation n_i. On the output side, the signal passes through two circulators which isolate the device from noise from the HEMT amplifier at 3 K.

The noise photon occupation n_i of a coaxial cable microwave mode, at a plate i, with frequency ω is given by

$$\begin{aligned} n_i(\omega) &= \frac{n_{i-1}(\omega)}{A_i} + \frac{A_i - 1}{A_i} n_{\text{BE}}(T_i, \omega), \\ n_{\text{BE}}(T, \omega) &= \frac{1}{e^{\frac{\hbar\omega}{k_B T}} - 1} \end{aligned} \tag{88}$$

where $n_{i-1}(\omega)$ is the occupation for the plate with the next higher temperature, $n_0 = n_{\text{BE}}(293\,\text{Kelvin}, \omega)$, A_i is the attenuation at the plate i with temperature T_i, such that for an attenuator with value -10 dB, $A = 10$.

The noise temperature of modes coming towards the device from the input is then $n_{\text{th,in}} < 0.108$, which is only an upper bound as additional attenuation in the cabling and connectors has not been taken into account. For modes coming towards the device from the output wiring, the noise is given by the 50 Ω Johnson noise in the isolator $n_{\text{th,out}} \simeq 0$. The thermal occupancy of the resonator n_{th} is the sum of the occupancy of each thermal bath to which the resonator is coupled to, weighted by the coupling to each bath. Since the device is over-coupled to the feedline, the average occupancies of modes coming towards the device from both directions is a good approximation of the resonator occupancy: $n_{\text{th}} < 0.05 \ll 1/2$. The output of the device is wired to the input of the VNA after being amplified with a high-electron-mobility transistor (HEMT) amplifier at ∼4 Kelvin, and a room-temperature amplifier.

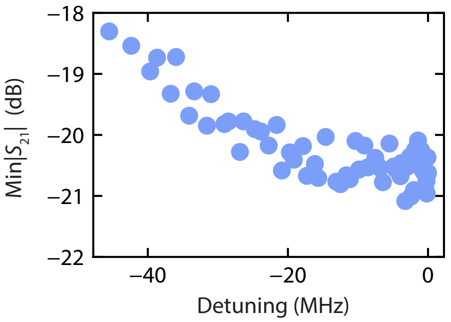

Supplementary Fig. 13. **Minimum of transmission as a function of detuning.** The detuning is defined as the difference between the circuit's resonance frequency – modified by the magnetic flux traversing the SQUID loop – and the resonance frequency used in the rest of the data presented in this work.

B. Frequency-dependence of damping

By sweeping the current in a coil located under the device we can change the static magnetic field traversing the device. Through this field, we are able to tune the Josephson inductance of the SQUID and hence the resonance frequency of the circuit. In the data presented in this paper, we operate at the first order magnetic field insensitive point of the SQUID - also known as flux "sweet-spot". In Supplementary Fig. 13, we show our analysis of the data presented in Ref. [5], where S_{21} is recorded as the flux through the SQUID is changed. This data set is acquired at a power of -138 dBm, far below the power at which the apparent nonlinear damping manifests. We find a change in the depth of the resonance peak as a function of detuning from the sweet-spot. However, the detuning required to match the change induced by power far exceeds any change in resonance frequency occurring through the Kerr nonlinearity. Indeed, the shift induced by driving the Kerr oscillator remains under 2 MHz (see Supplementary Fig. 3) but the minimum changes by 4 dB, whereas the changes in Min$|S_{21}|$ shown in Supplementary Fig. 13 in the detuning range of a few MHz remains smaller than 1 dB, dominated by experimental noise. This means for example that any frequency-dependence in the measurement port admittances as seen from the resonator is not responsible for the non-linear damping discussed in this work.

---

[1] S. E. Nigg, H. Paik, B. Vlastakis, G. Kirchmair, S. Shankar, L. Frunzio, M. Devoret, R. Schoelkopf, and S. Girvin, Black-box superconducting circuit quantization, Phys. Rev. Lett. **108**, 240502 (2012).

[2] M. F. Gely and G. A. Steele, Qucat: quantum circuit analyzer tool in python, New J. Phys. **22**, 013025 (2020).

[3] U. Vool and M. Devoret, Introduction to quantum electromagnetic circuits, Int. J. Circuit Theory Appl. **45**, 897 (2017).

[4] L. S. Bishop, *Circuit quantum electrodynamics*, Ph.D. thesis, Yale University (2010).

[5] S. Yanai and G. Steele, Observation of enhanced coherence in josephson squid cavities using a hybrid fabrication approach, arXiv preprint arXiv:1911.07119 (2019).

[6] M. F. Gely, G. A. Steele, and D. Bothner, Nature of the lamb shift in weakly anharmonic atoms: From normal-mode splitting to quantum fluctuations, Phys. Rev. A **98**, 053808 (2018).

[7] R. A. Horn and C. R. Johnson, *Matrix analysis* (Cambridge University Press, 1985) pp. 146–147.

[8] P. Virtanen, R. Gommers, T. E. Oliphant, M. Haberland, T. Reddy, D. Cournapeau, E. Burovski, P. Peterson, W. Weckesser, J. Bright, S. J. van der Walt, M. Brett, J. Wilson, K. J. Millman, N. Mayorov, A. R. J. Nelson, E. Jones, R. Kern, E. Larson, C. J. Carey, İ. Polat, Y. Feng, E. W. Moore, J. VanderPlas, D. Laxalde, J. Perktold, R. Cimrman, I. Henriksen, E. A. Quintero, C. R. Harris, A. M. Archibald, A. H. Ribeiro, F. Pedregosa, P. van Mulbregt, and SciPy 1.0 Contributors, SciPy 1.0: Fundamental Algorithms for Scientific Computing in Python, Nat. Methods **17**, 261 (2020).

[9] B. Brock, J. Li, S. Kanhirathingal, B. Thyagarajan, W. F. Braasch, M. Blencowe, and A. Rimberg, Nonlinear charge- and flux-tunable cavity derived from an embedded cooper-pair transistor, Phys. Rev. Appl. **15**, 044009 (2021).

[10] P. Degenfeld-Schonburg, C. Navarrete-Benlloch, and M. J. Hartmann, Self-consistent projection operator theory in nonlinear quantum optical systems: A case study on degenerate optical parametric oscillators, Phys. Rev. A **91**, 053850 (2015).

[11] M. J. Powell, An efficient method for finding the minimum of a function of several variables without calculating derivatives, Comput. J. **7**, 155 (1964).

[12] W. Verstraelen and M. Wouters, Gaussian quantum trajectories for the variational simulation of open quantum-optical systems, Applied Sciences **8**, 10.3390/app8091427 (2018).

[13] C. Navarrete-Benlloch, E. Roldán, Y. Chang, and T. Shi, Regularized linearization for quantum nonlinear optical cavities: application to degenerate optical parametric oscillators, Opt. Express **22**, 24010 (2014).
[14] P. Drummond and D. Walls, Quantum theory of optical bistability. i. nonlinear polarisability model, J. Phys. Math. Gen. **13**, 725 (1980).
[15] K. Kheruntsyan, Wigner function for a driven anharmonic oscillator, J. Opt. B: Quantum Semiclass. Opt. **1**, 225 (1999).
[16] C. Gardiner and P. Zoller, *The quantum world of ultra-cold atoms and light book II: The physics of quantum-optical devices* (Imperial College Press, 2015).
[17] D. P. DiVincenzo and J. A. Smolin, Nonlinear spectroscopy of superconducting anharmonic resonators, New J. Phys. **14**, 013051 (2012).
[18] C. H. Meaney, H. Nha, T. Duty, and G. J. Milburn, Quantum and classical nonlinear dynamics in a microwave cavity, EPJ Quantum Technol. **1**, 7 (2014).
[19] A. Isar, A. Sandulescu, and W. Scheid, Phase space representation for open quantum systems within the lindblad theory, Int. J. Mod. Phys. B **10**, 2767 (1996).
[20] M. Stobińska, G. Milburn, and K. Wódkiewicz, Wigner function evolution of quantum states in the presence of self-kerr interaction, Phys. Rev. A **78**, 013810 (2008).
[21] M. Oliva and O. Steuernagel, Quantum kerr oscillators' evolution in phase space: Wigner current, symmetries, shear suppression, and special states, Phys. Rev. A **99**, 032104 (2019).
[22] I. Katz, R. Lifshitz, A. Retzker, and R. Straub, Classical to quantum transition of a driven nonlinear nanomechanical resonator, New Journal of Physics **10**, 125023 (2008).
[23] W. F. Braasch Jr, O. D. Friedman, A. J. Rimberg, and M. P. Blencowe, Wigner current for open quantum systems, Phys. Rev. A **100**, 012124 (2019).
[24] N. H. McCoy, On the function in quantum mechanics which corresponds to a given function in classical mechanics, Proceedings of the National Academy of Sciences of the United States of America **18**, 674 (1932).
[25] J. R. Johansson, P. D. Nation, and F. Nori, Qutip: An open-source python framework for the dynamics of open quantum systems, Comput. Phys. Commun. **183**, 1760 (2012).
[26] J. Johansson, P. Nation, and F. Nori, Qutip 2: A python framework for the dynamics of open quantum systems, Comput. Phys. Commun. **184**, 1234 (2013).
[27] We note that, in the absence of thermal fluctuations, $n_{\mathrm{th}} < 0.05$, as we expect in our experiment, the mismatch between the resonance detuning $\Delta = K\alpha^2$ as contemplated by a classical description of the damped driven Kerr oscillator and its approximated counterpart value of Supp. Eq. (59) that derives from our quantum description, is lesser than a 4% for $P_{\mathrm{in}} \leq -124$ dBm. Thus, using Supp. Eq. (59) instead, entails almost no change in the steady-state of the Wigner currents with its overall features remaining the same.
